# Advancements and Innovations in Low-Temperature Hydrogen Electrochemical Conversion Devices Driven by 3D Printing Technology

**DOI:** 10.1007/s40820-025-01907-w

**Published:** 2025-09-08

**Authors:** Min Wang, Xiuyue Wang, Enyang Sun, Zhenye Kang, Fan Gong, Bin Hou, Gaoqiang Yang, Mingbo Wu, Feng-Yuan Zhang

**Affiliations:** 1 College of New Energy, China University of Petroleum (East China), Qingdao, 266580 People’s Republic of China; 2 School of Marine Science and Engineering, Hainan University, Haikou, 570228 People’s Republic of China; 3 Department of Energy and Power Engineering, College of Mechanical and Vehicle Engineering, Hunan University, Changsha, 410082 People’s Republic of China; 4 Department of Mechanical, Aerospace & Biomedical Engineering, University of Tennessee, Knoxville, Knoxville, TN 37996 USA

**Keywords:** 3D printing, Hydrogen, Proton exchange membrane fuel cells, Water electrolyzers

## Abstract

3D printing, as a versatile additive manufacturing technique, offers high design flexibility, rapid prototyping, minimal material waste, and the capability to fabricate complex, customized geometries. These attributes make it particularly well-suited for low-temperature hydrogen electrochemical conversion devices—specifically, proton exchange membrane fuel cells, proton exchange membrane electrolyzer cells, anion exchange membrane electrolyzer cells, and alkaline electrolyzers—which demand finely structured components such as catalyst layers, gas diffusion layers, electrodes, porous transport layers, and bipolar plates. This review provides a focused and critical summary of the current progress in applying 3D printing technologies to these key components. It begins with a concise introduction to the principles and classifications of mainstream 3D printing methods relevant to the hydrogen energy sector and proceeds to analyze their specific applications and performance impacts across different device architectures. Finally, the review identifies existing technical challenges and outlines future research directions to accelerate the integration of 3D printing in next-generation low-temperature hydrogen energy systems.

## Highlights

- Outlines 3D printing methods and their benefits in fabricating complex components for low-temperature hydrogen devices.
- Summarizes current applications in fuel cells and electrolyzers, highlighting recent progress in hydrogen energy.
- Explores future directions and challenges, offering insights into trends and opportunities in hydrogen-related systems.

## Graphical abstract

**Figure:**
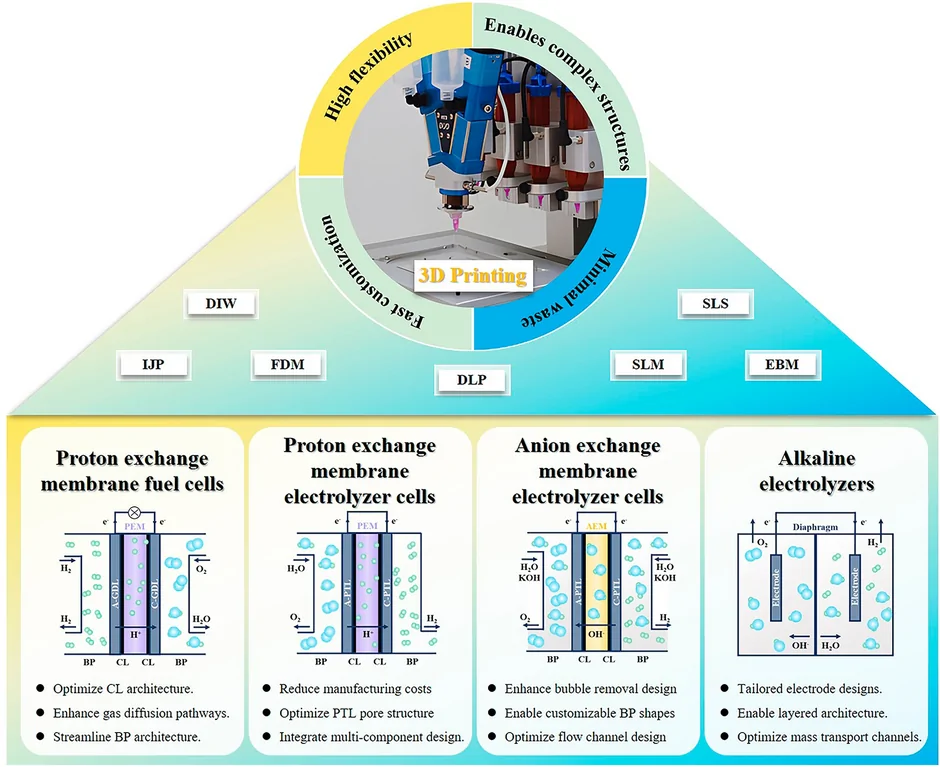

## Introduction

Energy transition is a global trend, and finding more environmentally friendly and low-carbon energy solutions is an inevitable path [1, 2]. Hydrogen, termed the "ultimate energy" of the twenty-first century, represents a genuinely green and low-carbon clean energy source and has gradually become one of the pivotal carriers in the global energy transition [3–6]. Water electrolyzers and fuel cells, as two key low-temperature devices for hydrogen conversion, have rapidly developed over the past few years [7–10]. Water electrolyzers, significant for hydrogen production, can be integrated with renewable energy generation technologies. During the hydrogen production process, they essentially produce no greenhouse gases, making them a crucial pathway for generating green hydrogen [11]. In the realm of fuel cells, particularly proton exchange membrane fuel cells (PEMFCs), they are noted for their high conversion efficiency and rapid response time even at low temperatures, thereby excelling in specific operational contexts, such as heavy-duty trucks on fixed routes [12–15].

3D printing technology represents an advanced method of object fabrication, characterized by its ability to construct three-dimensional objects through the successive layering of materials [16]. Compared to traditional subtractive manufacturing techniques, the unique advantages of 3D printing have garnered significant attention within the hydrogen energy sector [17, 18]. Primarily, this technology offers remarkable flexibility and customization [19]. Utilizing computer-aided design software, users can precisely engineer and construct objects of various shapes, sizes, and structures to meet specific requirements [20, 21]. This capability provides expansive opportunities for innovation in hydrogen-related industries, such as designing flow field configurations for water electrolyzers and bipolar plates in fuel cells [22]. Additionally, 3D printing is more efficient and cost-effective relative to conventional manufacturing methods, which typically require the creation of molds or tools and involve multiple production steps and extended durations. By enabling direct object production from digital models, 3D printing reduces both time and costs, effectively lowering the costs associated with hydrogen production [23]. Furthermore, 3D printing is resource-conserving and environmentally friendly, as it minimizes waste production by manufacturing on demand, thereby significantly reducing resource wastage [24, 25]. The high adaptability of 3D printing technology within the hydrogen energy field is poised to accelerate the industrialization and widespread application of hydrogen technologies [26, 27].

**Fig. 1:**
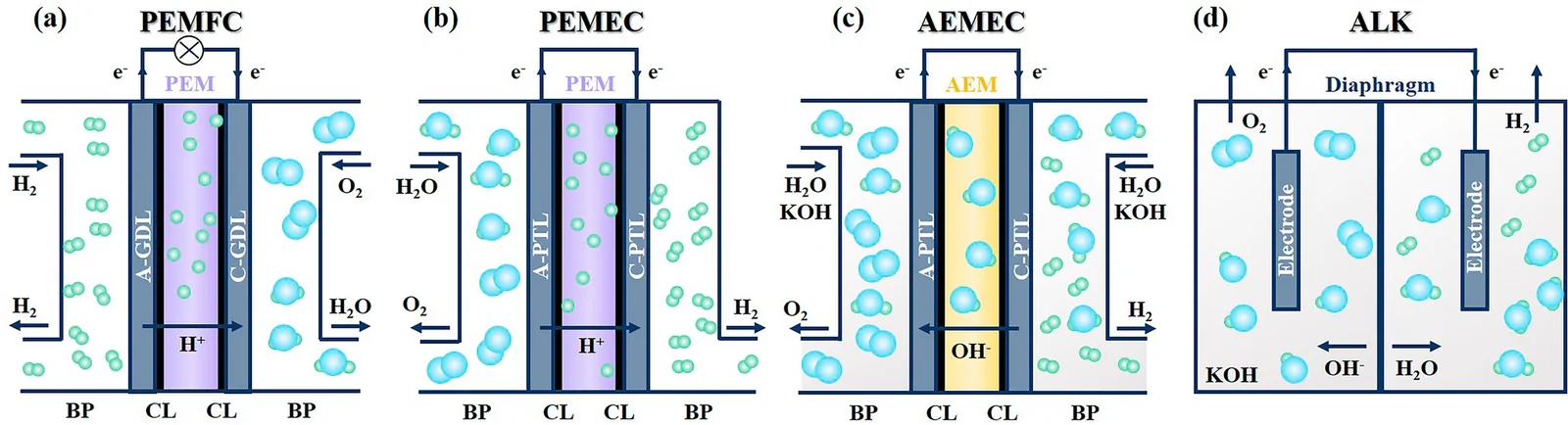
Schematic diagram of **a** PEMFC, **b** PEMEC, **c** AEMEC, and **d** ALK. Reproduced with permission from Ref. [9]. Copyright 2022, Tsinghua University Press

As illustrated in Fig. 1, the fundamental structures and components of PEMFCs and various types of water electrolyzers are depicted. In the PEMFCs, the main components include the bipolar plates (BP), gas diffusion layers (GDL), catalyst layers (CL), and proton exchange membrane (PEM). Except for the fabrication of the PEM, the other components can be produced using 3D printing technology. The water electrolyzers are primarily divided into proton exchange membrane electrolyzer cells (PEMECs), alkaline electrolyzers (ALK), and anion exchange membrane electrolyzer cells (AEMECs). Components amenable to integration with 3D printing technology include the electrodes, BP, and porous transport layers (PTL). For both PEMFCs and water electrolysis systems, the operating temperature generally does not exceed 100 °C. The low-temperature environment not only favors the long-term use of 3D-printed components but also imposes relatively lower requirements for the materials’ high-temperature resistance and corrosion resistance, thus providing a broader range of material choices for 3D-printing technologies. Table 1 provides a succinct comparison and introduction to the reaction equations, as well as the advantages and disadvantages of the PEMFCs and different types of water electrolyzers.

**Table 1:** Introduction to Fuel cells and Various Water Electrolyzers

Category	Fuel cells	Water Electrolyzers		
	PEMFC	PEMEC	ALK	AEMEC
Electrolyte	PEM	PEM	KOH/NaOH	AEM
Typical Catalyst	Pt/C	Ir; Ru; Pt	Ni; Co; Mn	Ni; Co; Fe
Anode Reaction	$\frac{1}{2}O_{2} \left( g \right) + 2H^{ + } + 2e^{ - } \to H_{2} O\left( l \right)$	$H_{2} O\left( l \right) \to \frac{1}{2}O_{2} \left( g \right) + 2H^{ + } + 2e^{ - }$	$2OH^{ - } \to \frac{1}{2}O_{2} \left( g \right) + H_{2} O\left( l \right) + 2e^{ - }$	$2OH^{ - } \to \frac{1}{2}O_{2} \left( g \right) + H_{2} O\left( l \right) + 2e^{ - }$
Cathode Reaction	$H_{2} \left( g \right) \to 2H^{ + } + 2e^{ - }$	$2H^{ + } + 2e^{ - } \to H_{2} \left( g \right)$	$2H_{2} O\left( l \right) + 2e^{ - } \to H_{2} \left( g \right) + 2OH^{ - }$	$2H_{2} O\left( l \right) + 2e^{ - } \to H_{2} \left( g \right) + 2OH^{ - }$
Overall Reaction	$\frac{1}{2}O_{2} \left( g \right) + H_{2} \left( g \right) \to H_{2} O\left( l \right)$	$H_{2} O\left( l \right) \to \frac{1}{2}O_{2} \left( g \right) + H_{2} \left( g \right)$	$H_{2} O\left( l \right) \to \frac{1}{2}O_{2} \left( g \right) + H_{2} \left( g \right)$	$H_{2} O\left( l \right) \to \frac{1}{2}O_{2} \left( g \right) + H_{2} \left( g \right)$
Advantages	Zero pollution High work efficiency Good dynamic response	Safe and pollution-free Fast response time	Mature technology Low cost	Uses non-precious metal catalysts Strong adaptability
Disadvantages	Higher cost Insufficient technology penetration	Higher cost Durability needs improvement	Corrosion and contamination issues High maintenance cost Long response time	Anion exchange membrane preparation technology needs breakthroughs

Jiao et al. [12] discuss the development trends of critical components of PEMFCs, with particular emphasis on the integration of the GDL and BP through 3D printing technologies. Traditional manufacturing methods face challenges in achieving such integration, but combining these with 3D printing holds promise for realizing this concept. Currently, the main issues with this integration are the poor compatibility of materials and insufficient structural precision. An integrated structure can effectively reduce interface resistance and simplify assembly processes. This approach has been previously explored in water electrolyzers, with experimental results affirming its feasibility [28]. Moreover, the configuration and gradient of devices can be effortlessly controlled through computer-aided design and printed without the need for molds [29]. In PEMFCs, such structural innovations can positively impact water and gas management [30], aiding in the separation and transfer of bubbles in water electrolyzers [31]. Particularly with the continuous advancements in 3D printing technology, its compatibility with the hydrogen energy sector is expected to reach unprecedented levels.

This article starts by introducing the working principles of 3D printing technology, further delving into its extensive applications in PEMFCs and water electrolyzers. The classification in PEMFCs includes the CL, BP, and GDL, based on components fabricated through 3D printing. In the case of water electrolyzers, the classification comprises electrode, BP, and PTL. Finally, the paper summarizes the challenges encountered by 3D printing technology in this emerging field and forecasts the future development trends and prospects of these technologies.

## Overview of 3D Printing Technology

3D printing technology, characterized by its operational flexibility, high production efficiency [32, 33], and access to a broad range of materials, has been extensively applied across various domains in recent years, including but not limited to bio-intelligent systems and energy conversion fields [34]. A variety of 3D printing techniques, such as direct ink writing (DIW), inkjet printing (IJP), fused deposition modeling (FDM), digital light processing (DLP), selective laser sintering (SLS), selective laser melting (SLM), and electron beam melting (EBM), have been significantly developed. These technologies have also found applications in the domain of hydrogen energy, thereby laying a foundational framework for advancing the role of 3D printing in the hydrogen sector [26, 35, 36]. They have been instrumental not only in improving electrode design and component fabrication but also in optimizing the overall architecture of electrochemical cells. By leveraging 3D printing, researchers can enhance fluid dynamics and boost operational efficiency—both critical to the performance of hydrogen energy devices. This section focuses on the primary 3D printing methods employed in hydrogen energy applications, with an emphasis on raw materials, core processes, and key influencing factors. The advantages and disadvantages of different 3D printing technologies are shown in Table 2.

**Table 2:** Comparison of 3D Printing Technologies for Low-Temperature Hydrogen Electrochemical Conversion Devices

Technology Type	Applicable Components	Technology Features	Advantages	Disadvantages
DIW	CL/GDL/PTL/Electrode	Deposits material as continuous fibers via a nozzle to build complex structures	High material flexibility Customizable properties Complex structures possible	Slow print speed Limited resolution Material preparation complex
IJP	CL	Precisely deposits material droplets via printheads to form uniform and controllable structures	High resolution Material saving Fast layer deposition	Limited material types Nozzle clogging risk
FDM	BP	Extrudes molten material layer by layer to build parts	Low cost Wide material range	Low resolution Poor surface finish Limited mechanical strength
DLP	CL/GDL/PTL/Electrode	Uses light to cure resin layer by layer, achieving high precision	High precision Fast printing speed Good surface quality	Limited material types High equipment cost Post-curing required
SLS	CL/GDL/PTL/Electrode	Uses a laser to sinter powder materials, eliminating the need for support structures	Wide material range No support structures High mechanical strength	Material waste Post-processing needed
SLM	CL/GDL/PTL/Electrode	Melts metal powder with a laser to build high-density complex structures	High-density parts Complex structures High mechanical properties	Low resolution Post-processing required
EBM	CL/GDL/PTL/Electrode	Melts metal powder with an electron beam in a vacuum for rapid forming	Rapid forming High energy efficiency	High equipment cost Size limitations

### Direct Ink Writing

**Fig. 2:**
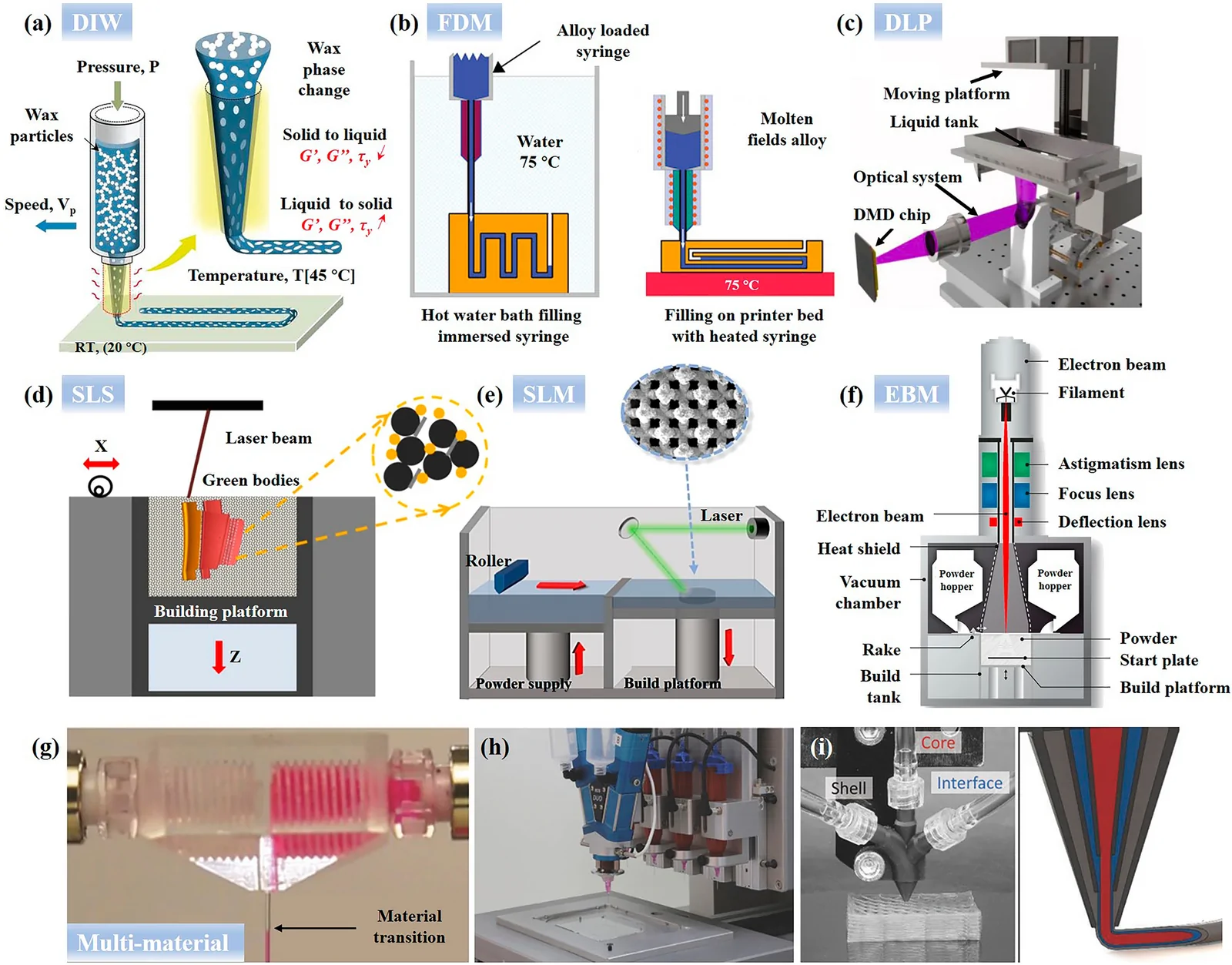
**a** Schematic diagram of DIW process. Reproduced with permission from Ref. [39]. Copyright 2022, Wiley–VCH GmbH. **b** FDM utilizing a medium container for printing. Reproduced with permission from Ref. [48]. Copyright 2022, Wiley–VCH GmbH. **c** DLP experimental equipment. Reproduced with permission from Ref. [33]. Copyright 2021, Elsevier. **d** SLS printing process. Reproduced with permission from Ref. [25]. Copyright 2021, Elsevier. **e** SLM printing process. Reproduced with permission from Ref. [71]. Copyright 2023, Elsevier. **f** EBM experimental equipment. Reproduced with permission from Ref. [78]. Copyright 2018, Elsevier. **g** Multi-material printing device with two independently controlled injection pumps. Reproduced with permission from Ref. [86]. Copyright 2022, Wiley–VCH GmbH. **h** Picture of the equipment with four pressure-driven syringe dispensers and a two-component mixing dispenser. Reproduced with permission from Ref. [87]. Copyright 2022, Wiley–VCH GmbH. **i** Schematic diagram of a multi-core shell nozzle. Reproduced with permission. Reproduced with permission from Ref. [88]. Copyright 2018, Wiley–VCH GmbH

DIW represents a quintessential extrusion deposition technology in 3D printing (Fig. 2a), applicable to a myriad of materials for fabricating structurally complex 3D objects [37–40]. This technique involves the precise, layered stacking of flowable pastes to craft objects with desired shapes and functionalities, finding extensive applications in the field of hydrogen energy [41]. Typically, pastes with high viscoelasticity and self-supporting characteristics are chosen to ensure that the material maintains its intended shape upon deposition to the substrate without succumbing to gravitational deformation or leveling [42]. The rheological behavior of the paste, which refers to its deformation and flow properties under external forces or strains, serves as a primary characterization method in DIW. Rheological parameters are crucial for controlling changes in the paste, impacting both the printability and the final structure significantly [43]. The yield stress (σ_y_) is the threshold at which the paste begins to flow and deform; controlling σ_y_ is vital for ensuring appropriate rheological properties during printing. An optimal σ_y_ facilitates fine structural control throughout the printing process [44]. Furthermore, the storage modulus (G’) and the loss modulus (G’’) reflect the paste's elasticity and viscosity, respectively, both of which influence the rheological behavior during the DIW process. A higher G’ helps maintain the structure’s shape, while an appropriate G’’ ensures sufficient plasticity and deformability. Viscosity (η) affects the resistance to flow of the paste, directly impacting the printing speed and the resolution of the structure. By adjusting η, one can achieve the desired flowability while maintaining structural integrity [45]. Understanding these parameters is instrumental in modulating the rheological performance during the printing process, ensuring the quality and fidelity of the desired shapes.

### Inkjet Printing

IJP technology capitalizes on the capabilities of inkjet heads to eject liquid ink onto paper or other substrates, facilitating the selective layer-by-layer deposition of liquid ink to form patterns or three-dimensional models while precisely controlling their structure and composition [46]. This technique is extensively utilized across various domains, including printing, drawing, marking, and the application of functional coatings. In comparison to alternative printing methods, IJP is distinguished by its high precision in ink deposition, which ensures the accuracy of the printed dosage by controlling the volume emitted from the nozzles. Moreover, IJP offers considerable flexibility regarding substrates; ink formulations can be printed on a diverse range of materials [47] and can be directly employed for patterned manufacturing of CL on PEM. However, there are inherent limitations associated with IJP, such as the relatively high cost of inks and the complexity of their formulation. The printing quality is contingent upon the state of the ink, and the technology's slower speed during high-resolution printing considerably hinders its development in the hydrogen energy sector.

### Fused Deposition Modeling

FDM is a 3D printing technology based on the extrusion of molten filament materials (Fig. 2b) [48]. This process is characterized by its simplicity, user-friendliness, and low cost, making it particularly suitable for rapid prototyping of thermoplastic resins [49, 50]. In the FDM process, the rapid prototyping system feeds the filament to the print head, where the material is heated above its melting point. Under the precise control of a computer, the print head moves along a predetermined path on the build plate, extruding the molten filament to form the desired shape [51]. Variations in the parameters of the FDM printing process significantly influence the mechanical properties of the final product [52]. Studies have demonstrated that the nozzle temperature primarily affects the flowability of the extruded material, and its precise control is crucial for enhancing the interlayer shear strength [53]. Parameters such as the fill angle and layer thickness substantially impact tensile strength, flexural strength, and impact resistance, with layer thickness being a predominant factor affecting tensile and flexural performance [54]. Adjustments in fill density and nozzle diameter also significantly influence the porosity of the product, while fill density affects surface roughness and nozzle diameter impacts contour shape accuracy [55]. By carefully controlling these process parameters, it is possible to fabricate FDM-printed products with superior mechanical properties, meeting the specific requirements of different devices in the field of hydrogen energy.

### Digital Light Processing

DLP printing technology, as depicted in Fig. 2c, leverages projected ultraviolet (UV) light to initiate cross-linking in low molecular weight photosensitive resins, resulting in the formation of high molecular weight polymers [56, 57]. The UV light, processed by a digital micromirror device (DMD), projects the cross-sectional images of the model onto the surface, thereby printing the layered images of the designed model layer-by-layer until the entire three-dimensional structural model is completed. In this technology, the intensity and stability of the UV light directly impact the printing speed and quality. High-intensity UV sources typically enhance printing speed, while the stability of the UV source aids in maintaining consistent print outcomes [58]. The resolution of the DMD profoundly influences the minimum precision achievable by the printer. While striving for accuracy, efficiency considerations must also be balanced, as higher precision often necessitates longer print times. The selection of the DMD resolution thus requires a trade-off between accuracy and efficiency [59]. DLP technology also imposes specific requirements on printing materials. The photosensitive resin used must be compatible with the specific wavelength of the UV light and possess appropriate curing characteristics. Furthermore, the material needs to exhibit good flow properties to ensure timely coverage of the print area and filling of the model, presenting a challenge in material selection [60]. Compared to other photolithographic printing methods such as stereolithography (SLA), DLP can achieve higher structural precision [57]. However, maintaining uniformly stable flow characteristics of the paste in practical printing processes remains challenging, affecting precision stability [61]. Nevertheless, as technology continually advances, the shortcomings of DLP are gradually being overcome and improved. The technology has found applications in various fields, including the custom production of medical implants [62], fabrication of optical components [63], and printing of porous electrodes [64].

### Selective Laser Sintering

SLS, occasionally referred to as direct metal laser sintering (DMLS) [65], employs a computer-controlled laser that sequentially scans and sinters powdered material, constructing cross-sections of an object (Fig. 2d). During this process, the laser selectively fuses powder particles along the contour of the current layer. The thermal energy causes the particles to bond, gradually accumulating to form a solid object [66, 67]. After each layer is completed, the build platform descends by one layer thickness to accommodate a new layer of powder, and this procedure is repeated until the entire three-dimensional model is constructed. This layer-by-layer advancement is similar to that of DLP, but SLS differs in that it utilizes a laser as the light source, which offers broader material versatility. From the perspective of SLS principles, various materials such as plastics, metals, and ceramics can serve as raw materials for selective laser sintering if they can be bonded upon exposure to laser heat to form the necessary shapes [68]. Additionally, SLS does not require support structures, allowing materials to bond more robustly and enhancing mechanical stability [69]. In the realm of hydrogen energy, titanium is a crucial metal and can be used as raw material for bipolar plates. The combination of SLS technology with titanium alloy powders facilitates the manufacturing of components with complex geometrical shapes and fine structures [70], thereby driving innovation in the production of critical components such as bipolar plates and advancing hydrogen technology.

### Selective Laser Melting

SLM technology, as depicted in Fig. 2e, employs a laser to melt metal powders layer by layer, thereby constructing three-dimensional objects [71]. The system leverages a laser beam to control the scan paths meticulously, targeting specific regions of the metal powder. The energy emitted by the laser instantaneously melts or partially melts the powder in the targeted areas. Upon the completion of a scan, the build platform descends by a minuscule distance, followed by the application of a new layer of metal powder. The laser then scans this new layer, melting the powder to gradually form the cross-sections of the three-dimensional object. This layer-by-layer construction process is repeated until the entire object is fabricated [72]. In SLM processes, the porosity and impurities within each layer are minimal, which facilitates the homogenization of defects [73]. Post-processing steps, such as the removal of unmelted powder, surface treatment, and heat treatment [74], are generally required after printing to meet the final physical and mechanical property specifications.

### Electron Beam Melting

EBM represents an advanced metal 3D printing technology (Fig. 2f). This manufacturing technique involves utilizing an electron beam as the heat source to construct three-dimensional objects through the sequential printing and stacking of metal powders [75]. The process closely mirrors SLM, yet EBM operates around an electron beam. The high energy of the electron beam facilitates rapid melting of the metal powders, thus enhancing overall production efficiency [76]. In practical applications, titanium and its alloys serve as crucial materials within the aerospace sector [77]. Aerospace components often require a combination of high mechanical strength and precision. EBM technology meets these demands and aligns well with large-scale production [78, 79] The high-temperature environment of the melting process enables precise printing of complex geometrical shapes in high-temperature metal alloys such as titanium alloys [80], offering an outstanding solution for the manufacture of aircraft and spacecraft. Beyond aerospace, EBM also demonstrates unique value in fields such as the medical and energy sectors. In the medical domain, EBM is employed to fabricate orthopedic implants. The high precision and customizability of this technology ensure that the implants are optimally adapted to the patient's bone structure [81–83]. In the energy sector, EBM holds potential for manufacturing parts with low residual stress and high strength, thereby enhancing the performance and longevity of energy equipment [84].

In addition to the aforementioned techniques that have been practically applied in the field of hydrogen energy involving the printing of singular materials, considerable attention has been directed towards multi-material and multi-scale hybrid printing technologies [85]. These include, but are not limited to, multi-nozzle printing, hybrid jet printing, extrusion-based multi-material printing, and multi-material laser sintering techniques. Multi-material 3D printing technology enables the use of multiple materials in a single printing process, significantly enhancing design flexibility and functionality. As shown in Fig. 2g–i, different modes of multi-material printing can be achieved by controlling the nozzle structure: alternating printing of multiple materials [86], mixed printing of multiple materials [87], and materials in a mutually encapsulated state [88]. The rapid development of multi-material 3D printing technology allows for the replication of the diverse characteristics of complex components, offering new possibilities for creating sophisticated and high-performance 3D-printed structures [89]. Moreover, multi-scale 3D printing technology integrates both micro and macro 3D printing, allowing for the creation of structures at both microscopic and macroscopic scales to meet different structural precision requirements. In the integration of water electrolyzers and fuel cells, there is a demand for components to utilize materials of varying qualities and precision. Should this technology become widespread, it would inevitably promote integrated design advancements.

## Application of 3D Printing Technology in Proton Exchange Membrane Fuel Cells

### Fabrication of Catalyst Layer via 3D Printing Technology

**Fig. 3:**
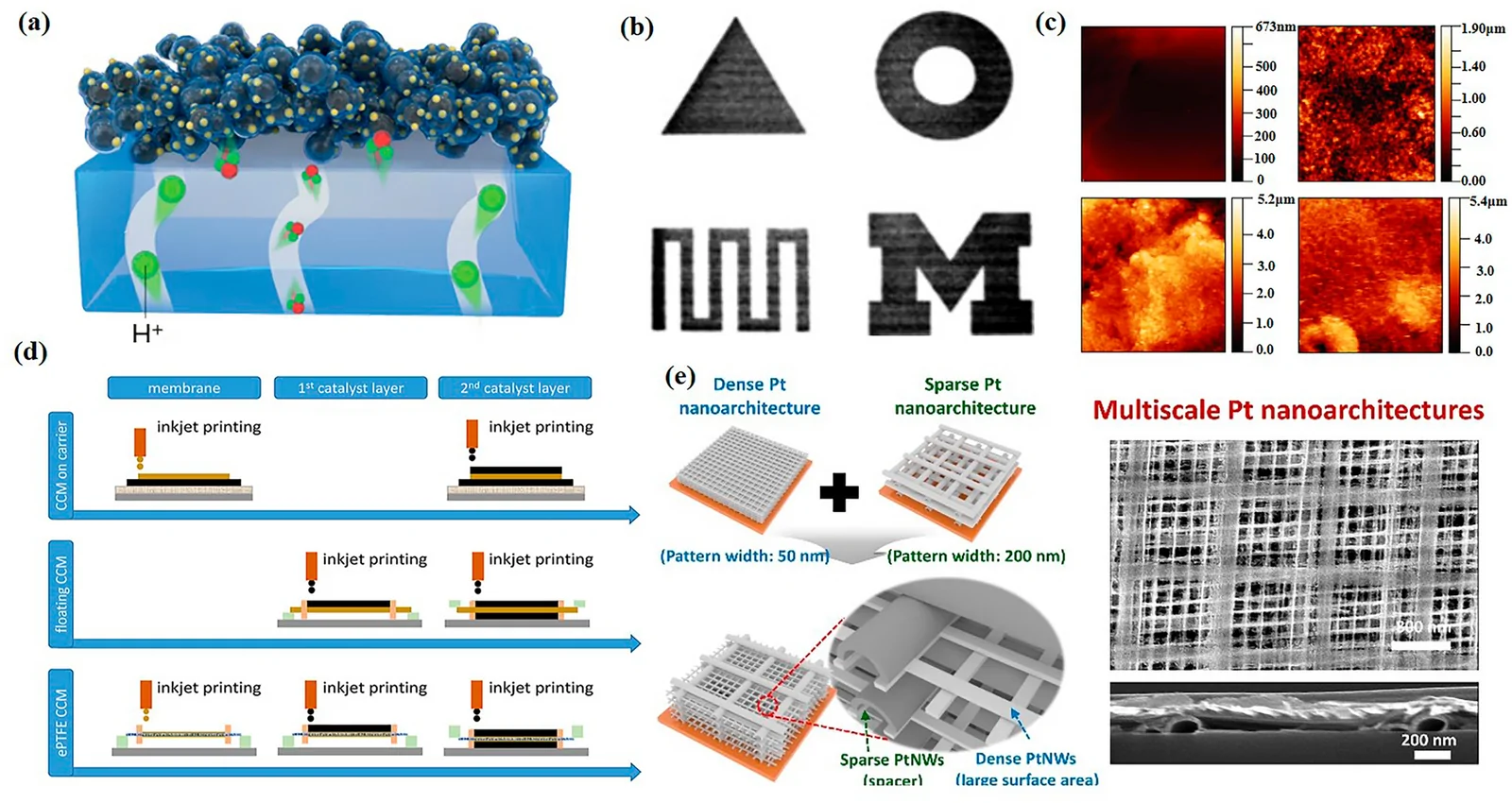
**a** Schematic representation of a CL on a membrane. Reproduced with permission from Ref. [12]. Copyright 2021, Springer Nature. **b** Schematics of various patterns of CL printed using the IJP method. Reproduced with permission from Ref. [101]. Copyright 2007, Elsevier. **c** AFM images of a Nafion membrane before (top left) and after (top right) printing and of carbon paper before (bottom left) and after (bottom right) printing. Reproduced with permission from Ref. [102]. Copyright 2019, Wiley–VCH GmbH. **d** Schematic of three different CCMs manufactured using IJP. Reproduced with permission from Ref. [103]. Copyright 2022, Elsevier. **e** Schematic of a multi-scale platinum nanowire structure. Reproduced with permission from Ref. [106]. Copyright 2018, AAAS

As illustrated in Fig. 3a, the CL is identified as a pivotal component within the membrane electrode assembly (MEA), serving as the primary site for electrochemical reactions in PEMFCs [90]. Specifically, when the catalyst is directly coated onto the GDL, it forms a gas diffusion electrode (GDE) [91–93], whereas when the catalyst is coated onto both sides of the PEM, it forms a catalyst-coated membrane (CCM) [94]. Hydrogen undergoes a hydrogen oxidation reaction (HOR) at the anode CL, producing protons that are subsequently transported toward the cathode CL. These protons participate in the oxygen reduction reaction (ORR) with oxygen and electrons at the catalyst active site [95, 96]. Due to the slow kinetics of the ORR, the cathode CL is currently the primary focus of research aimed at enhancing the performance of PEMFCs [97, 98]. For instance, some studies have focused on reducing oxygen transport resistance in the cathode CL through an ordered design, which facilitates faster oxygen diffusion to the three-phase boundary (TPB), enhancing its participation in the electrochemical reaction [99]. Moreover, the development of novel, low-cost catalysts is prioritized due to the high costs associated with the catalysts [100]. Despite numerous studies on non-precious metal catalysts, platinum on carbon remains the most effective and commercially viable catalyst. Therefore, reducing the platinum loading has become an essential trend towards cost reduction in PEMFC development. Taylor et al. [101] were the pioneers in utilizing IJP to deposit CL onto the GDL, forming patterned GDE, as depicted in Fig. 3b. They successfully printed catalyst ink onto GDL and demonstrated that varying the deposition patterns of CL could be achieved through controlled printing. IJP allows for the deposition of small volumes of aqueous-based catalyst inks with precision reaching picoliter levels. In their work, the catalyst loading was reduced to 0.020 mg_Pt_ cm^−2^, with a platinum utilization exceeding 16 W mg^−1^_Pt_. Furthermore, by employing IJP for layer-by-layer deposition of catalysts with varying platinum concentrations—higher near the membrane and lower further from it—they showed that gradient CL performs better than uniformly distributed CL when the total loading is similar. Subsequently, Deiner et al. [102] used IJP to prepare CCM, GDE, and a bilayer of CCM and GDE, demonstrating that GDE prepared by thermal pressing achieved a power density of 1.067 W cm^−2^, surpassing that of MEA based on CCM (0.579 W cm^−2^). Moreover, the superiority of IJP-prepared GDE was further validated through electrochemical impedance spectroscopy and microstructural analysis. As shown in Fig. 3c, atomic force microscopy (AFM) revealed that the roughness (R_q_) of Nafion increased by an order of magnitude post-printing, whereas only a minimal increase was observed in the R_q_ of the carbon paper. This difference in deposition fidelity between Nafion and carbon paper resulted in divergent performance outcomes. Differing from the aforementioned approaches, Willert et al. [103] explored three methodologies for 3D printing of CCM functional layers using IJP, as shown in Fig. 3d: the first method involved depositing a polymer layer and a second CL on top of a base layer of slot-die-coated CL; the second involved depositing two CL layers on a commercial membrane; and the third used an enhanced PTFE (ePTFE) layer as a substrate, on which Nafion and CL were deposited on both sides. They found that MEA based on ePTFE substrates exhibited a peak power density 15% higher than traditional MEA, highlighting its superior performance. Currently, IJP technology has matured in its application within PEMFCs CL, offering significant advantages in reducing catalyst loading and controlling microscale morphology compared to traditional CL fabrication methods. Additionally, the design of patterned CL structures has also entered the research domain [104, 105], providing new perspectives on the potential applications of IJP technology in CL development.

In the realm of 3D printing, IJP is not the sole technology applied to the fabrication of CL for PEMFCs. A notable development was undertaken by Jung et al. [106], who engineered a three-dimensional tailored architecture of platinum nanostructures demonstrating superior ORR activity compared to conventional Pt/C when tested in a rotating disk electrode (RDE) system. As depicted in Fig. 3e, the research utilization of nano-transfer printing to create platinum nanowire catalysts with feature dimensions of 50 and 200 nm, respectively. However, in single-cell configurations, the former exhibited a propensity for flooding at high current densities due to elevated mass transfer resistance, while the latter underperformed due to its lower porosity. To address these challenges, the authors proposed a multiscale arrangement of platinum nanowires, alternating between dense and sparse building blocks, which enhanced the maximum power density by 43% over Pt/C electrodes. This work signifies an innovative stride in leveraging 3D printing for constructing three-dimensional CL structures, particularly in designing ordered sub-micrometer-scale pore channels, a capability beyond the current scope of IJP technology. Nevertheless, there remains room for improvement. For instance, the absence of ionomers might increase the proton transfer impedance between the membrane and CL, although adding ionomers could potentially raise the mass transfer impedance. It is therefore suggested that enhancing the hydrophilicity of the platinum nanowire CL could better meet the requirements for proton transport.

In addition to inkjet and nano-transfer printing, several other additive manufacturing techniques have emerged for catalyst layer and support fabrication in PEM systems. For example, hierarchical nanoporous gold electrodes have been realized via extrusion-based 3D printing followed by dealloying, producing multiscale porosity—from macroscale architectures (10–1000 μm) to nanoscale ligaments (30–500 nm)—that markedly enhance mass transport and catalytic accessibility in both liquid and gas-phase reactions [107]. SLM-based direct metal printing has also enabled the creation of mechanically robust, hollow metallic scaffolds with tunable pore geometries; when these supports are coated in situ with NiCo_2_S_4_ nanoneedles, they exhibit low overpotentials, small Tafel slopes, and outstanding oxygen evolution reaction (OER) performance at current densities up to 100 mA cm^−2^, with improved bubble release and corrosion resistance [108]. Furthermore, SLM has facilitated the fabrication of monolithic metallic electrodes incorporating internal reactant delivery channels, resulting in ~ 40% enhanced hydrogen oxidation kinetics by alleviating mass transport limitations [109]. These advances demonstrate the diverse capabilities of emerging 3D printing methods—each offering unique strengths in architectural design, mechanical integrity, and integrated transport features—and broaden the toolkit for engineering high-performance catalyst layers in PEMFCs.

Whether utilizing IJP for the deposition of CL or constructing CL through platinum nanowires, both approaches demonstrate significant advantages in enhancing the utilization of platinum catalysts. However, due to the ultra-thin thickness and complex pore structure of CL, the application of 3D printing in this context remains in a nascent stage. Among the methods employed, IJP, despite being widely used, shows no significant improvements in the performance of CL, primarily due to its inability to enhance structural design. Looking ahead, the orderly preparation of CL represents a future trend [12]. Compared to traditional CL, the orderly CL can enhance active sites, thereby significantly reducing the load of precious metals. 3D printing, controlled by predetermined computer programs, aligns well with the concept of orderly preparation of CL. With the rapid development in types and precision of 3D printing, the construction of orderly CL through this technology may increasingly come into the purview of researchers.

### Fabrication of Bipolar Plates Utilizing 3D Printing Technology

BP represents one of the most compatible components for integration with 3D printing technology within PEMFC systems [110, 111]. Leveraging 3D printing enables the customized fabrication of BP, allowing for the flexible design of complex structures to enhance cell performance and effectively address design challenges that are difficult to overcome with traditional manufacturing techniques [22]. As pioneers in integrating these two technologies, Bourell et al. [112] conducted a detailed study on the effect of chopped carbon fiber volume fraction on the strength and conductivity of graphite BP fabricated via laser sintering. They found that the flexural strength of the finished parts increased from 35 MPa to nearly 50 MPa with the addition of carbon fibers. This finding confirms the viability of combining these methodologies from an intrinsic property’s perspective. In addition to graphite, a common BP material in PEMFCs, Lee et al. [113] explored printing polymers using FDM technology to develop BP structures with optimized shapes. They reported a three-type flow channel design with an accuracy of 88.8%. However, neither Bourell et al. nor Lee et al. progressed beyond the BP printing optimization stage; neither study applied the printed BP in an actual PEMFC system. Also, in FDM technology, to address the issue of poor conductivity in 3D-printed polymer BP, Jang et al. [114] successfully enhanced the performance by sputtering a silver coating onto the polymer BP surface. This modification enabled the BP to achieve an open-circuit voltage of 0.96 V in PEMFC testing. D. Gould and colleagues [111] employed DMLS to produce titanium alloy plates with embedded flow fields, subsequently applying these in PEMFCs. Although the performance discrepancies from expected outcomes persisted, this further substantiates the feasibility of 3D printing in the fabrication of PEMFC components. As 3D printing technology continues to advance, improvements in component precision and surface smoothness are expected to meet, if not exceed, anticipated performance levels. The rapid evolution of 3D printing technology promises enhanced flexibility, efficiency, and performance in PEMFC systems, potentially driving innovation and progress in PEMFC technology [115].

**Fig. 4:**
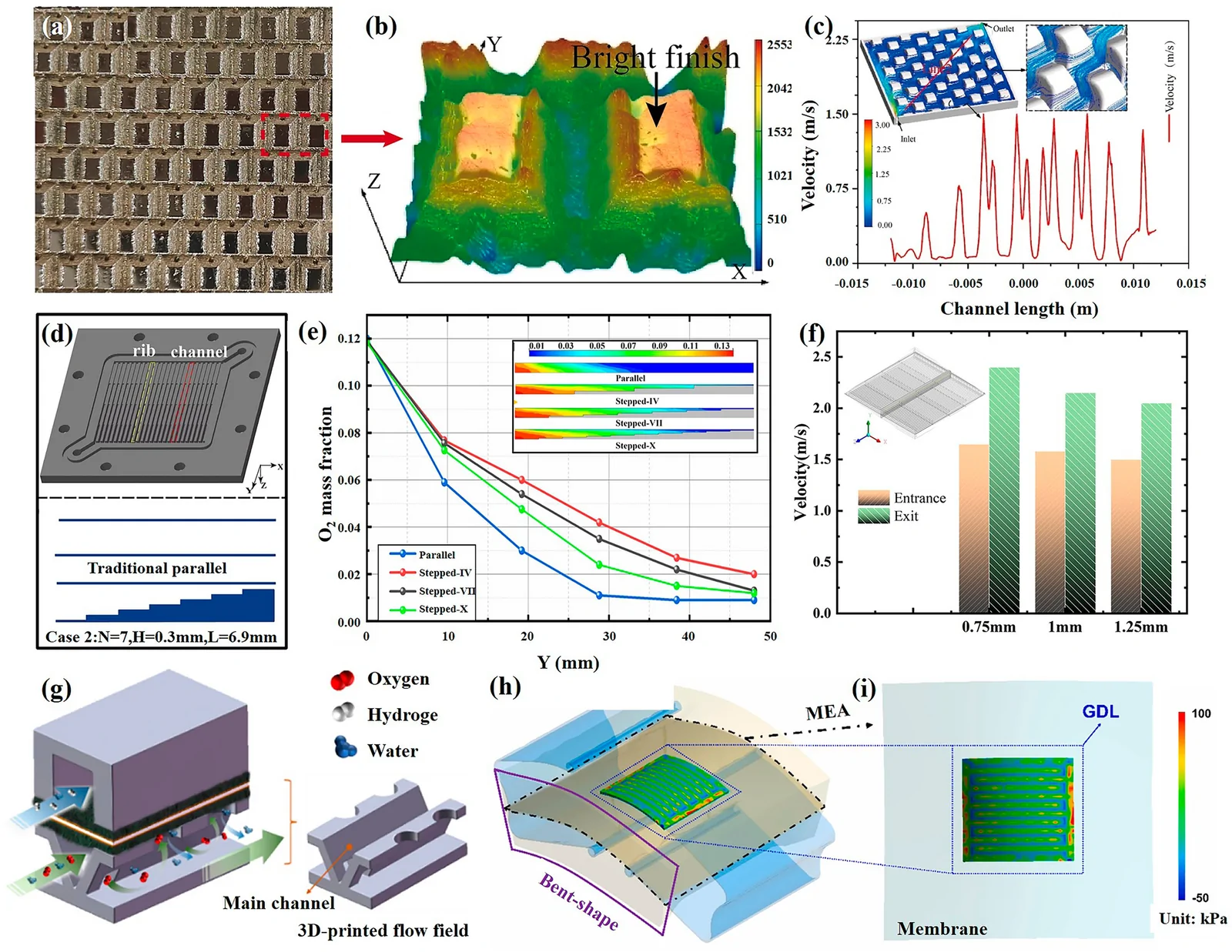
**a** Macroscopic morphology of the post-polishing 3D-III surface. **b** Localized three-dimensional view of the 3D-III surface following polishing. **c** Gas flow rates at the cathode side GDL/CL interface of the 3D-III structure. Reproduced with permission from Ref. [118]. Copyright 2023, Elsevier. **d** Comparative diagram of traditional parallel flow fields and various structured tertiary flow fields. **e** Oxygen mass fraction in the middle channel of the flow field along the y-axis and its distribution in the y–z cross-section (voltage = 0.55 V): y-axis and y–z axis cross-section. **f** Gas flow rates at the inlet and outlet of a single cell with varying tread widths at a voltage of 0.55 V. Reproduced with permission from Ref. [22]. Copyright 2024, Elsevier. **g** Schematic of the novel flow field. Reproduced with permission from Ref. [30]. Copyright 2022, American Institute of Chemical Engineers **h** Schematic representation of a PEMFC with a curved flow field plate. i Top-down 2D finite element analysis of the PEMFC curved flow field plate. Reproduced with permission from Ref. [121]. Copyright 2022, Elsevier

In recent years, 3D printing technology has garnered significant attention, with marked improvements in precision that facilitate the fabrication of complex three-dimensional structures [116]. In PEMFC systems, three-dimensional flow fields, compared to traditional 2D flow fields, can leverage angular gradients to enhance the entry of reactant gases into the CL, thereby improving the uniformity of gas–liquid distribution. Huang et al. [117] successfully fabricated bipolar plates with grid flow fields and obstacle flow fields using SLM technology. The results showed that the bipolar plates fabricated using SLM technology had high dimensional accuracy (error ≤ 3%) and surface quality (average R_a_ = 0.5 μm). The SLM process has significant advantages in the fabrication of bipolar plates with complex flow field structures. Performance data for individual cells can be obtained quickly, providing experimental data support for subsequent structural optimization. Wang et al. [118] employed SLM printing technology to design and fabricate a three-dimensional flow field bipolar plate composed of hollow ramp units. The polished real-life images and their 3D profiles are shown in Fig. 4a, b. The results showed that the maximum power density generated by 3D-III was 451.4 mW cm^−2^. In PEMFCs, the flow rate of oxygen directly influences the distribution of oxygen and water within the cell, as demonstrated in Fig. 4c, which depicts the oxygen flow rate distribution at the cathode side GDL/CL interface when using 3D-III. Due to the perforations in the elevated areas of 3D-III, the reactant gases have a longer residence time in the flow field, allowing more extensive diffusion into the CL. This is beneficial for enhancing the oxygen concentration distribution on the CL. The red curve in the figure represents the change in oxygen flow rate along the Line 2 direction; at the inlet, the lower flow rate prevents water from being carried into the CL with the gas stream, reducing the water content at the CL, whereas at the outlet, an increased oxygen flow rate facilitates the timely expulsion of product water.

Lu and colleagues [22] combined simulation with experimental methods to study the mass transfer issues in three-dimensional flow field bipolar plates, using SLM technology to construct 3D step flow fields with varying step widths (Fig. 4d). At 0.55 V, the measured power density of the Stepped-IV cell reached as high as 672.7 mW cm^−2^, which is about 26% higher than the conventional parallel flow field. To more intuitively and concretely illustrate the impact of flow field structure on the transport performance of reactive gases, the authors processed computational fluid dynamics (CFD) data to obtain the oxygen mass fraction along the y-axis direction of the intermediate channels and their distribution on the yz-plane cross-section, as shown in Fig. 4e. The oxygen mass fraction in different flow field channels decreases progressively from the inlet to the outlet, with a notable oxygen deficiency occurring at the outlet of the parallel flow field channel. In contrast, the stepped flow field significantly alleviates this oxygen deficiency problem. When the step size was set to 0.75 mm, the inlet and outlet flow velocities reached approximately 1.64 m s^−1^ and 2.39 m s^−1^, respectively, which was significantly better than when the step size was set to 1 and 1.25 mm. This is attributed to the smaller cross-sectional area of narrower step channels, which enhances the flow velocity of gases, strengthens convective effects, and effectively improves gas–liquid mass transfer performance (Fig. 4f). Local oxygen concentration under the flow channel ribs is lower, and water flooding is more severe, which also affects PEMFC performance [119]. Addressing this issue, Chen et al. [30] utilized metal 3D printing technology to intricately modify traditional parallel and serpentine flow fields by creating some ribs as hollow structures and incorporating auxiliary channels (Fig. 4g). This modification increased local oxygen concentrations under the ribs and enhanced drainage capabilities, thereby improving cell performance under high current densities.

Should PEMFCs exhibit the capacity for flexible bending, their applicability would be substantially extended [120]. Such versatility would render them suitable for use as power sources in portable applications, including wearable electronics and foldable smartphones. Traditional fabrication methods pose significant challenges in producing flexible PEMFCs. In contrast, utilizing the versatility of 3D printing technology appears feasible for realizing this concept. Yoo et al. [121] innovatively adopted 3D printing technology and successfully printed a bendable flow field plate (Fig. 4h) using a flexible, rubber-like, translucent photosensitive polymer called TangoPlus. They creatively applied it to PEMFCs, thereby improving performance. Subsequent finite element analysis elucidated the performance enhancement: as the curvature of the PEMFCs increases, the compressive stress at the MEA escalates, leading to a significant reduction in ohmic resistance and charge transfer resistance. In addition, durability tests were conducted to investigate the irreversible degradation of the MEA caused by flexible fuel cells, although the test period was relatively short at only 12 h. However, comparing the first cycle with the last cycle, the rate of irreversible performance degradation was approximately 0.34%, which is negligible. The successful fabrication and application of flexible flow field plates hold extraordinary significance for broadening the application spectrum of PEMFCs.

The design of the flow field on the BP exerts a substantial impact on the performance of PEMFCs. An optimal flow field design can enhance gas transport, reduce mass transfer polarization, and improve the cell's water management capabilities [122]. In recent years, researchers have developed a variety of structurally distinct flow field configurations, including but not limited to stepped flow field [123], three-dimensional wavy flow field [124, 125], biomimetic leaf vein flow field [126], and biomimetic honeycomb flow field [127]. Theoretically, these configurations can facilitate water drainage in PEMFCs; however, due to manufacturing constraints, certain flow field designs have remained solely in the numerical simulation phase. Three-dimensional printing technology represents a viable solution to this limitation. It allows for the production and in situ testing of various theoretical flow field structures, thereby uncovering operational issues that simulations fail to address. This capability enables subsequent modifications and advances in BP development.

### Fabrication of Gas Diffusion Layers via 3D Printing Technology

**Fig. 5:**
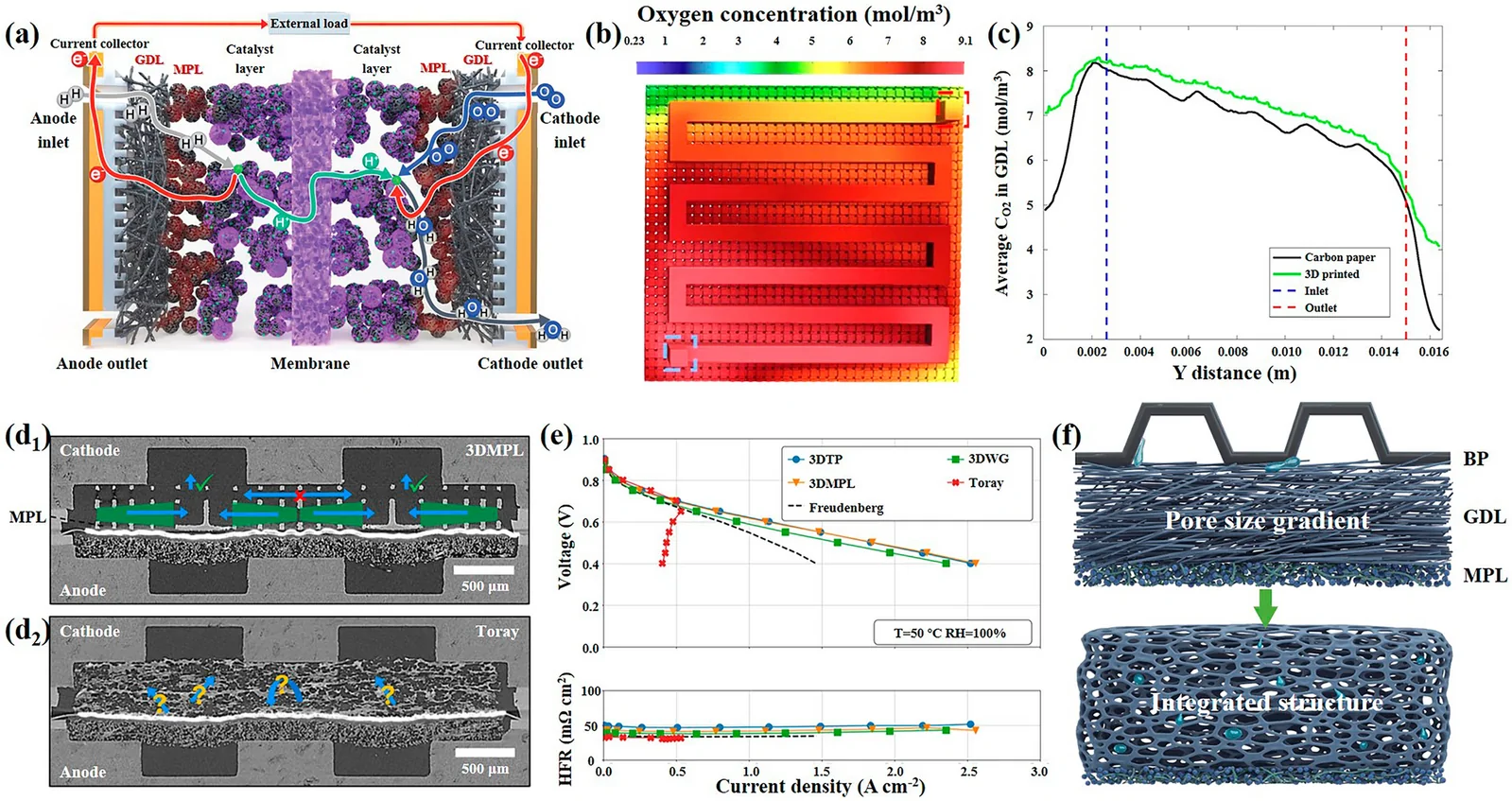
**a** Schematic diagram of typical GDL and MPL in PEMFC. Reproduced with permission from Ref. [100]. Copyright 2021, Wiley–VCH GmbH. **b** Flow field view. **c** 3D GDL (in green) and carbon paper (in black) along the Y-direction, showcasing the average oxygen concentration in the X-direction within the GDL. Reproduced with permission from Ref. [135]. Copyright 2022, Elsevier. **d**_**1**_ PEMFC assembly with printed water guide GDL featuring an MPL 3DMPL at the cathode. **d**_**2**_ PEMFC assembly with Ref.erence Toray GDL at the cathode side. **e** Polarization curves and HFR of the tested samples obtained at T = 50 °C and RH = 100%. Reproduced with permission from Ref. [136]. Copyright 2025, American Chemical Society. **f** Developmental trends of GDL: from traditional separated structures to integrated BD and GDL structures. Reproduced with permission from Ref. [12]. Copyright 2021, Springer Nature

GDL is recognized as a pivotal component in PEMFCs, bridging the CL and flow fields and facilitating the transport channels for gas phases [96, 128–130]. As depicted in Fig. 5a, GDL typically consist of a macroporous layer and a microporous layer (MPL), but some GDL do not contain MPL. The macroporous layer principally provides structural support to the GDL, predominantly fabricated from carbon paper. In contrast, the MPL, characterized by smaller pore sizes, plays a crucial role in distributing the gas–liquid phases within the system, typically formed by mixing carbon black with hydrophobic materials through spraying or pressing techniques [131, 132]. However, due to the limited mechanical properties of carbon paper and its susceptibility to carbon corrosion [133], Arunkumar et al. [134] explored the fabrication of a carbon-free GDL using 3D printing technology. The authors employed SLS to ablate aluminum powder to serve as a GDL substrate, successfully producing alumina and manufacturing a uniform, defect-free film on its surface to enhance its conductive and thermal properties. Despite the compatibility of the aluminide's mechanical and electrical characteristics with the operational conditions of PEMFCs, a significant limitation is aluminum's propensity to oxidize in PEMFC environments, potentially damaging the membrane components with formed metal ions, thus questioning the feasibility of this approach.

Furthermore, Niblett and colleagues [135] pioneered the application of 3D-printed GDL in PEMFCs. They experimented with GDLs of varying microstructures, ultimately selecting those that did not deform post-carbonization for use in PEMFCs. Employing CFD, the authors investigated the ideal performance of oxygen distribution within the cathode flow field and the GDL (Fig. 5b). The results indicated a more uniform and effective distribution of oxygen across the flow field facilitated by the 3D GDL, with enhanced diffusion flux and convective flux towards the edges of the flow field, areas where diffusion limitations were markedly evident in traditional carbon paper. The oxygen concentration distribution data extracted from the GDL region, averaged along the X-axis from the inlet to the outlet, are presented in Fig. 5c. Within all areas of the cell, the oxygen concentration in 3D GDL (represented by the green line) was consistently higher than that in carbon paper materials (depicted by the black line). However, the application of 3D GDL in PEMFCs is not devoid of challenges, such as notably lower open-circuit voltages, attributed to the inability of 3D GDL to match the precision of commercial carbon papers, potentially causing damage to the membrane and consequently severe hydrogen permeation, significantly impacting the power density of the PEMFCs. Simultaneously, the larger pore size of the 3D GDL reduces the contact area with the CL by more than half, significantly increasing the contact resistance. These observations validate the feasibility of 3D printing in PEMFCs and highlight the need for continued research to ideally integrate these technologies. Recently, Dörenkamp et al. [136] have made significant progress in the 3D printing of GDL for use in PEMFCs. The authors addressed common water management issues in PEMFCs by using DLP technology to fabricate GDLs with different structures from photopolymerizable Phrozen Aqua-Gray 8 K resin, as shown in Fig. 5d_1_, d_2_, which illustrate the best-performing 3DMPL and the commercial Toray assembly structure. Concurrently, in-situ testing and advanced characterization simulations validated its unique role in water permeability guidance. Polarization curves and high-frequency resistance (HFR) test results both demonstrated the feasibility of applying 3DMPL (Fig. 5e). Compared to Toray’s poor performance under high humidity conditions, the performance of 3DMPL remained notably superior.

In PEMFCs, the mechanical properties, electrical conductivity, and gas–liquid transfer capabilities of the GDL present significant challenges. The performance of the GDL substantially impacts the power density of PEMFCs. Due to its role in facilitating gas–liquid transport, porosity is a critical parameter for the GDL [137]. However, higher porosity may compromise the contact between the GDL and the CL, often necessitating the addition of an MPL on the surface of the GDL as a form of structural optimization. In the future, modifications to the GDL structure, particularly gradient design, are anticipated to enhance gas supply and liquid expulsion, positively influencing the mass transport processes within the GDL [12]. Zhang et al. [138], in designing next-generation PEMFCs, noted that future directions for GDL development primarily involve gradation and integrated assembly, which can be perfectly achieved through 3D printing technology. With examples already present in water electrolyzers, the continuous development of 3D printing technology is believed to significantly propel the evolution of GDL in PEMFCs. Reducing the thickness of the component is further enhanced by utilizing 3D printing to integrate the GDL with the BP, as shown in Fig. 5f. This approach reduces overall thickness and eliminates contact resistance between the GDL and BP. Table 3 summarizes the typical applications of 3D printing in PEMFCs.

**Table 3:** Typical Applications of 3D Printing in PEMFCs

Component	Printing Methods	Characteristics of Application	Impact	Current Density @0.6 V	Year of Publication	References
CL	IJP	Graded CL	IJP applied to CL preparation IJP enables precise catalyst deposition, especially for ultra-low platinum	0.508 A cm^−2^ (80 °C, H_2_/O_2_)	2007	[101]
	SLM	Create intricate monolithic geometries	Promote mass transfer	/	2019	[109]
BP	DMLS	Titanium alloy BP with embedded flow channels	DMLS prints titanium alloy BP DMLS enables complex structures with no welds Rapid prototyping validation, but weight and flatness need improvement	0.987 A cm^−2^ (80 °C, H_2_/Air)	2015	[111]
	FDM	Silver-plated polymer BP	FDM printing ultra-light polymer BP A silver layer was sputtered on the polymer BP surface to enhance conductivity	0.450 A cm^−2^ (25 °C, H_2_/Air)	2022	[114]
	SLM	3D flow field with hollow slope units	SLM fabricates hollow slope flow fields 3D printing combined with CFD model validation	0.756 A cm^−2^ (50 °C, H_2_/Air)	2023	[118]
	SLM	3D step flow field	SLM combined with a 3D flow field New 3D flow field plates outperform conventional 2D plates Fewer steps and narrower width improve cell performance	1.123 A cm^−2^ (70 °C, H_2_/Air)	2024	[22]
GDL	SLS	3D-Printed Aluminum GDL	First attempt at SLS fabrication of non-carbon-based GDL	/	2016	[139]
	DLP	GDL with porous microstructures	3D printing of carbon-based GDL Controlled pyrolysis preserves microstructure	0.604 A cm^−2^ (60 °C, H_2_/ O_2_)	2022	[135]
	DLP	GDL with water permeability guidance function	3D-printed resin undergoes carbonization treatment The excellent performance of 3DMPL in PEMFCs validates its application prospects	1.127 A cm^−2^ (50 °C, H_2_/ Air)	2025	[136]

## Application of 3D Printing Technology in Water Electrolytic Cells

In the context of PEMFCs, the BP and GDL are predominantly fabricated from carbon materials due to the specific functional requirements and work environments. Commonly, graphite BP and carbon paper GDL are employed in PEMFCs. In contrast, the anodic environment of water electrolyzers, characterized by high voltage and an oxygen-rich atmosphere, demands materials with superior corrosion resistance, often resulting in the use of titanium-based BP and PTL with coatings [135]. Furthermore, there are structural differences between the components used in PEMFCs and those in water electrolyzers. To ensure uniform gas distribution and prevent leakage, the GDL in PEMFCs is typically designed to be homogeneous and dense, incorporating MPL; whereas, the PTL in water electrolyzers exhibits a more pronounced porous structure, which facilitates greater surface area and enhances gas–liquid transport channels. These material and structural variations dictate distinct approaches to integrating water electrolyzers with 3D printing technology compared to their application in PEMFCs.

Overall, 3D printing technology has been extensively applied in ALK, predominantly utilized for the fabrication of electrodes with various configurations. Its application in PEMEC and AEMEC has been relatively limited, with few implementations focused on the preparation of PTL and BP. Research on the complete fabrication of water electrolyzers via 3D printing remains comparatively sparse [140, 141]. In the actual process of water electrolysis, the separation of gases and the removal of bubbles are critical; addressing these challenges is difficult when only focusing on individual key components and necessitates specialized designs of the complete electrolysis cell. It is anticipated that 3D printing technology could resolve these issues. With the ongoing advancements and refinements in 3D printing technologies, it is expected that they will bring more innovation and breakthroughs to the field of water electrolysis, offering increased possibilities [142, 143].

### Fabrication of Electrodes via 3D Printing Technology

**Fig. 6:**
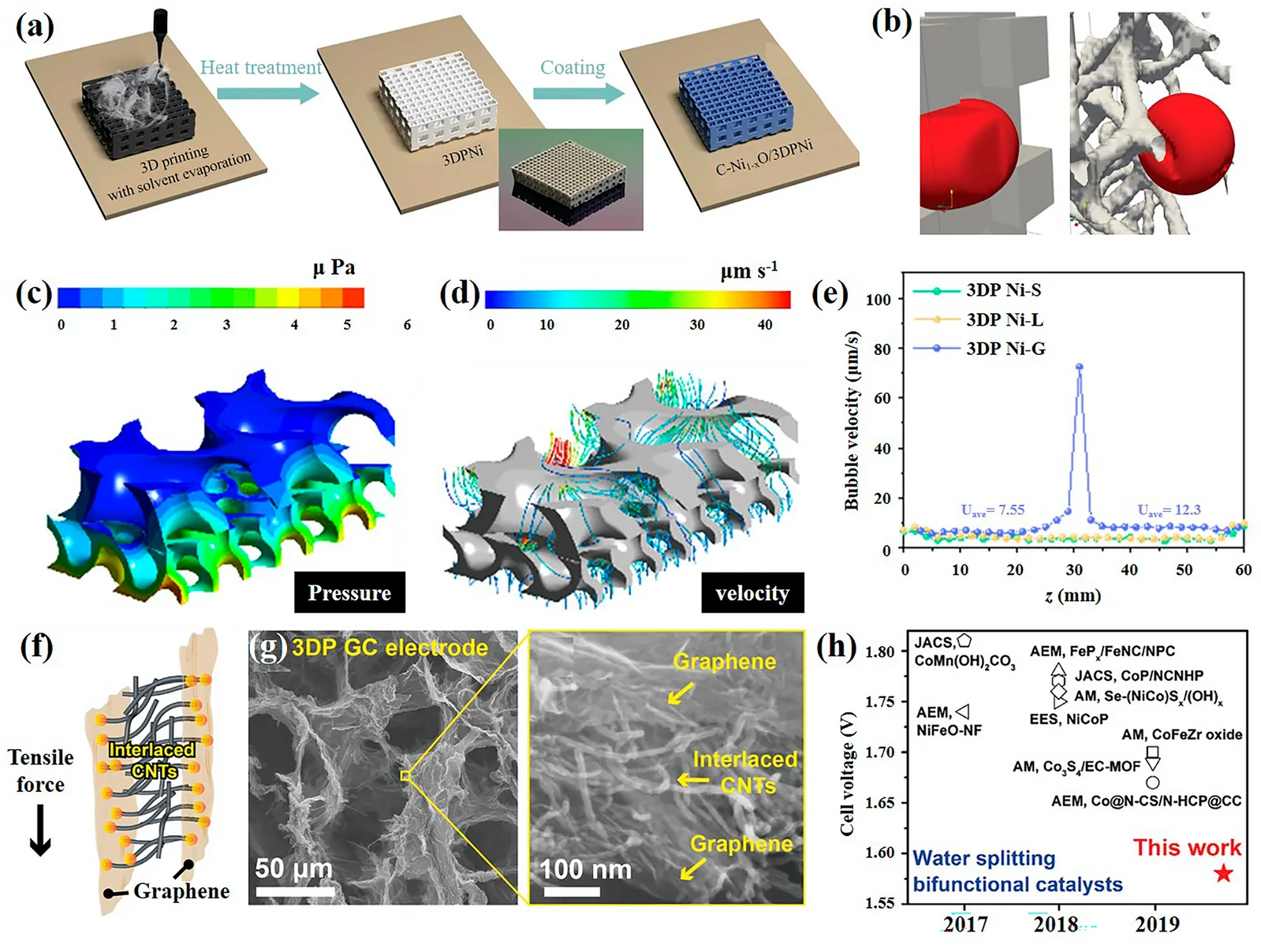
**a** Schematic illustration of the preparation of 3DPNi and its surface functionalization with C-Ni_1−x_O catalyst. **b** Schematic diagram of bubble transport simulation in 3DPNi and nickel foam. Reproduced with permission from Ref. [145]. Copyright 2020, Wiley–VCH GmbH. **c** Gradient-structured electrode internal pressure distribution. **d** Gradient-structured electrode internal bubble velocity distribution. **e** Average velocity of internal bubbles in 3DP Ni with different structures. Reproduced with permission from Ref. [147]. Copyright 2020, Wiley–VCH GmbH. **f** Schematic illustration shows the 3DP GC electrode can withstand the large bending force caused by the surface tension and buoyancy when immersed in water because of the bioinspired graphene/interlaced CNTs nanostructure. **g** SEM images of 3DP GC electrodes at different scales. **h** Comparison of the cell voltage of the 3DP GC/NiFeP-24L electrode with bifunctional water splitting catalysts reported in the literature at a current density of 30 mA cm^−2^. Reproduced with permission from Ref. [149]. Copyright 2020, Wiley–VCH GmbH

In the context of water electrolysis, the issue of gas–liquid two-phase mass transfer has consistently been a primary impediment to performance enhancement under high current densities [144]. During the electrolysis process, bubbles generated are likely to accumulate on the surface of the catalyst, which diminishes catalyst utilization, leads to an increase in ohmic resistance, and consequently restricts the hydrogen production rate [35]. Commonly, foamed nickel is employed as the electrode in ALK; its porous structure facilitates mass transfer, yet the disordered arrangement of the framework significantly hinders bubble migration. 3D-printed electrodes can be engineered to possess regular porous structures, which, while maintaining the intrinsic advantages of foam nickel, ensure rapid transfer of the gas–liquid two-phase system. Kou et al. [145] utilized DIW technology to fabricate 3D-printed nickel (3DPNi) electrodes with highly controllable periodic structures, effectively mitigating bubble aggregation. As depicted in Fig. 6a, the fabrication process of the 3DPNi electrode involves shaping the paste into a predetermined three-dimensional structure through a nozzle, followed by heat treatment and subsequent coating of the electrode surface with a C-Ni_1−x_O catalyst. Utilizing a high-speed camera, the authors developed a computational model based on the multiphase lattice Boltzmann method to study the migration process of individual bubbles within a porous medium. Within foamed nickel, the random distribution of pores leads to frequent collisions and deformations of bubbles, thereby increasing the propagation distance of the bubbles. In contrast, in periodic structures, the pore distribution is regular and the pore size larger, effectively shortening the bubble transfer distance (Fig. 6b). Following a similar approach, Xu et al. [146] employed DLP technology to fabricate gradient pore periodic structured 3D electrodes, which were then applied in a fully 3D-printed ALK. At a current density of 500 mA cm^−2^, these electrodes maintained performance without significant degradation over 1000 h, demonstrating the feasibility of this method.

Fu et al. [147] focused on the issue of bubble transfer during water electrolysis, developing a 3D-printed self-supporting metal electrode with Janus porosity. The gradient internal structure of the electrode creates an internal pressure differential that facilitates bubble migration. To intuitively explain the impact of the pore gradient structure on the internal bubble release of the electrode, simulations were conducted to examine the pressure distribution and velocity within the gradient structure. As depicted in Fig. 6c, the internal pressure in smaller pore structures is notably higher than that in larger pores, resulting in a significant internal pressure differential that drives hydrogen bubbles from smaller to larger pore structures. Figure 6d, e indicates that the average velocity of bubbles in the large pore structures on the upper side of the 3DP Ni-G electrode is approximately 12.3 μm s^−1^, which is 62.9% higher than that in the smaller pore structures on the middle and lower sides. This significantly accelerates the detachment of hydrogen from the electrode, demonstrating the positive effect of the gradient pore structure on bubble transport. Similarly, Rocha et al. [148] employed SLM technology to fabricate a 3D-printed electrode with a triply periodic minimal surface (TPMS), ideally having zero curvature to enhance gas expulsion. Through a combination of experimental and modeling approaches, the structural parameters of the TPMS were determined, contributing to the optimization of 3D geometric structures.

Differently from the focus on mass transfer processes, Peng et al. [149] have intensified research on the mechanical strength of 3D-printed electrodes. The authors innovatively incorporated a structure mimicking the setae on gecko feet, as illustrated in Fig. 6f, by interspersing one-dimensional carbon nanotubes among layers of two-dimensional graphene nanoplates during 3D printing. This addition enhances the interlayer friction, thereby augmenting the bending strength of the electrodes. Figure 6g depicts the biomimetic structure of graphene/interlaced carbon nanotubes on the walls of the 3DP GC electrode. As shown in Fig. 6h, compared to bifunctional water-splitting catalysts documented in related literature, the 3DP GC electrode exhibits superior performance, achieving a current density of 30 mA cm^−2^ at a low cell voltage of 1.58 V. The material was subsequently used as the cathode and anode in a two-electrode water-splitting cell. Large-area electrodes (L-3DP GC/NiFeP) were printed using 3D printing technology and installed in an H-type electrolytic cell to effectively separate and collect hydrogen and oxygen. This setup produced 1.34 mL of hydrogen and 2.64 mL of oxygen within 1200 s. These results highlight the scalability and practicality of 3D GC electrodes for large-scale applications.

In the realm of electrode manufacturing, besides the direct 3D printing of conductive materials to serve as electrodes, it is also a common approach to enhance insulating substrates through methods such as surface deposition and coating. Liu et al. [150] demonstrated the viability of a composite material by electrodepositing nickel on commercially available 3D-printed acrylonitrile butadiene styrene (ABS), followed by the deposition of a nickel–iron catalyst. The unmodified ABS displayed no OER activity, indicating that the deposition of the nickel–iron catalyst effectively enhances OER activity through surface modification. Meanwhile, Márquez et al. [151] employed SLS techniques to ablate nylon powder for electrode structuring. They sequentially deposited copper and nickel layers on the plastic electrode structures, followed by electroplating a nickel–iron coating in accordance with the literature. The resulting 3D electrodes exhibited favorable hydrogen evolution reaction (HER) and OER performances, as well as efficient bubble dispersion capabilities, thereby broadening the applicable scope of materials for 3D-printed electrodes.

In the field of electrolytic water electrode fabrication, 3D printing technology has demonstrated numerous successful applications. Predominantly, the controllability inherent to 3D printing has facilitated the creation of a diverse array of ordered electrode structures. The organized nature of these structures inherently advantages the promotion of mass transfer and bubble migration without substantially increasing costs, thus playing a pivotal role in enhancing the overall performance of water electrolysis. Looking forward, if advancements in simulation technology could be integrated, utilizing computational models to circumvent the time and resource expenditures associated with traditional trial-and-error approaches, it would be possible to identify electrode structures with optimal performance potential. Subsequently, the actual fabrication and performance validation of these structures through 3D printing could significantly enhance both the efficiency and accuracy of electrode design. This strategy, aimed at improving both the structure of electrodes and the overall performance of electrolytic water systems, represents a highly promising solution.

### Fabrication of Porous Transport Layers via 3D Printing

**Fig. 7:**
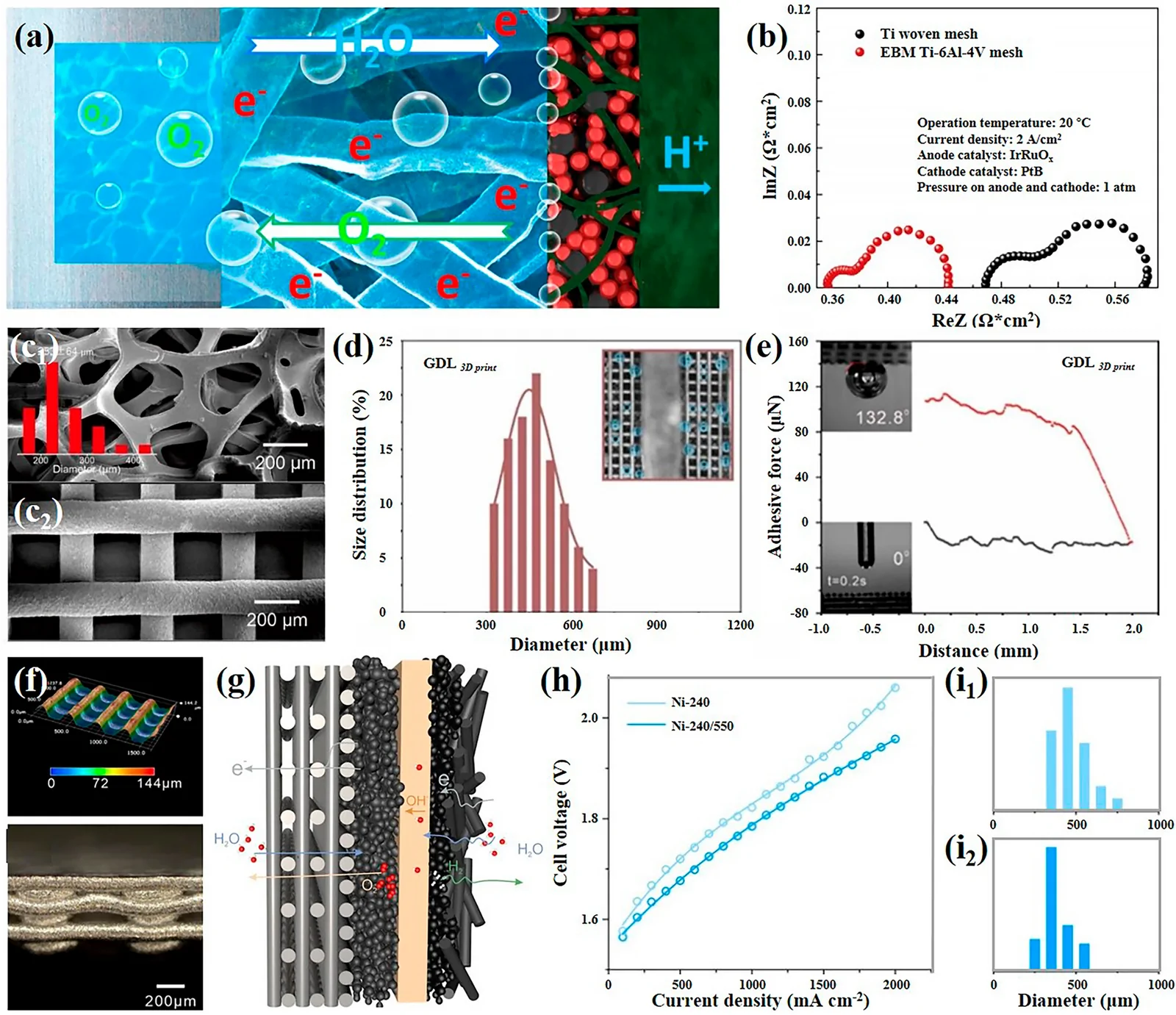
**a** Schematic representation of the anode-side PTL. **b** GEIS curves at a current density of 2 A cm^−2^ at room temperature using EBM Ti-6Al-4 V and Ti mesh as anode PTL in PEMEC. Reproduced with permission from Ref. [154]. Copyright 2016, Elsevier. **c**_**1**_ Optical microscope image of nickel foam. **c**_**2**_ Optical microscope image of the 3D-printed PTL. **d** Statistical distribution of oxygen bubble sizes released from the 3D-printed PTL operating at a current density of 1 A cm^−2^ in AEMEC. **e** Measurements of the adhesion force of O_2_ bubbles on the 3D-printed PTL, with insets showing comparative images of bubble contact angles and liquid contact angles. Reproduced with permission from Ref. [155]. Copyright 2023, Elsevier. **f** 3D contour map and physical cross-section image of Ni-240. **g** Schematic representation of a gradient 3D-printed Ni PTL used in the anode of AEMEC. **h** Performance comparison curves of Ni-240/550 versus Ni-240 in 80 °C pure water. **i**_**1**_ Statistical distribution of the size of O_2_ bubbles released by Ni-240. **i**_**2**_ Statistical distribution of the size of O_2_ bubbles released by Ni-240/550. Reproduced with permission from Ref. [31]. Copyright 2023, Wiley–VCH GmbH

In both the AEMEC and PEMEC devices, the PTL serves as a critical component within the electrolysis cell, as illustrated in Fig. 7a. The primary function of the PTL is to facilitate mass transfer between the gas–liquid phases across the CL and BP flow fields, supply electrons to all reaction sites, and ensure uniform heat distribution. An optimal PTL must meet the following criteria: 1) moderate porosity, 2) superior thermal and electrical conductivity, 3) high corrosion resistance, and 4) substantial mechanical stability. Utilizing 3D printing technology, complex three-dimensional designs can be converted from digital models into tangible objects. Specifically, for titanium, the most commonly used material in water electrolyzers, 3D printing enables the creation of PTL with controllable pore and structural configurations [152, 153]. This capability is instrumental in developing advanced PEMEC models with enhanced performance characteristics.

In the electrolysis of water, the potential at the anode is notably high, leading to an enrichment of oxygen. Given these conditions, the utilization of carbon-based materials, commonly employed as anode materials in PEMFCs, is impractical due to their susceptibility to degradation. Instead, corrosion-resistant metallic materials are more suitable for the design and manufacturing of PTL in water electrolyzers. On this basis, Mo et al. [154] employed EBM, a technique from the realm of 3D printing, to fabricate titanium-based anode PTLs, which were subsequently successfully implemented in PEMEC. The PTL fabricated via EBM showcased uniformly distributed square pores with smooth surfaces and consistent thickness, which significantly enhanced the contact interface and reduced the contact resistance with CL, as illustrated in Fig. 7b. Compared to titanium meshes with similar wire thickness and porosity, the EBM-fabricated PTL demonstrated an 8% improvement in performance. This success underscores the substantial potential of 3D printing technologies in the PEMEC sector. Further advancing the field, Huang et al. [155] utilized DIW technology to fabricate nickel-based PTL with three-dimensional periodic structures for use in AEMEC. Unlike traditional methods, which produce foamed nickel with tortuous gas–liquid diffusion channels as depicted in Fig. 7c_1_, leading to bubble accumulation and increased mass transfer resistance under high current densities, the DIW-fabricated PTL featured regularly arranged pores as shown in Fig. 7c_2_. This structural arrangement facilitated smaller bubble sizes during operation compared to those using foam nickel as PTL, allowing for more efficient expulsion of bubbles from the system and enhancing mass transfer efficiency, as shown in Fig. 7d. Additionally, the 3D-printed PTL exhibited enhanced hydrophilicity and oxygen-repelling properties, effectively reducing the adhesion force of bubbles on the PTL surface, as demonstrated in Fig. 7e, thereby decreasing the mass transfer resistance.

The pore size and distribution of the PTL play a crucial role in the mass transfer of gas–liquid phases within water electrolyzers. At high current densities, an increased porosity is necessary to enhance mass transfer rates and prevent high oxygen saturation, which can lead to bubble blockages and the creation of dead zones within the PTL. However, higher porosity levels reduce the contact area between interfaces, leading to increased electrical resistance and diminished overall electrochemical performance. Addressing this pivotal issue, Huang et al. [31] built upon previous work and utilized DIW to fabricate a series of periodically ordered Ni PTL with varying mesh sizes for research purposes. The ink used was prepared by a multi-solvent gradient evaporation method. The ink’s apparent viscosity drops sharply with increasing shear rate, showing shear-thinning and non-Newtonian behavior, which facilitates smooth nozzle flow. Additionally, the storage modulus exceeds the loss modulus, enabling the formation and stability of the printed structure. As illustrated in Fig. 7f, the 3D profile and optical microscopy images of the Ni PTL are presented for a mesh size of 240 μm. To concurrently tackle the challenges of mass transfer and interface contact, Huang et al. arranged Ni PTL of different mesh sizes in a gradient fashion. Figure 7g depicts a schematic cross-section of the gradient PTL. The experimentally designed Ni 240/550 gradient PTL achieved a current density of 2 A cm^−2^ at a cell voltage of 1.95 V, as shown in Fig. 7h, demonstrating superior battery performance. The comparison of bubble escape between Ni-240 and Ni 240/550, shown in Fig. 7i_1_, i_2_, indicates a significant reduction in bubble diameter after gradient modification, effectively enhancing the bubble release rate.

Given the demanding operational environment of the PTL in water electrolyzers, there is a high requirement for corrosion resistance. Titanium is the preferred substrate material due to its compatibility with such environments. However, manufacturing titanium into forms such as titanium felt or mesh through fine processing to ensure adequate mechanical strength and porosity significantly increases costs, which is not conducive to cost control in PEMEC. The utilization of technologies like SLM and SLS with titanium powder can achieve structured PTL with adjustable porosity and enhanced mechanical strength, reducing costs while improving two-phase transport within the PTL, thereby enhancing polarization performance. The main challenge currently faced by these technologies is achieving the required precision. PTL in PEMEC typically requires thicknesses at the sub-millimeter scale, while commercially available SLM and SLS technologies generally have resolutions around the millimeter scale. In addition to well-known metal printing techniques, recent advancements have been made through a collaboration between Mitsubishi Materials Corporation and Yokohama National University, using binder jetting 3D printing technology. This method uses pure titanium powder to successfully fabricate a novel titanium electrolytic electrode with a gradient dual-layer structure for use in PEMEC, capable of operating at current densities up to 4 A cm^−2^. This successful fabrication not only provides a new solution for precision issues but also opens up possibilities for overcoming the technological limitations of conventional metal 3D printing to support the development of hydrogen energy technologies. Emerging 3D printing technologies, such as binder jetting, which can achieve resolutions down to 10 µm, meet the basic requirements for devices in the hydrogen sector. Further exploration is needed to assess the compatibility of this technology with specific components.

### Fabrication of Bipolar Plates Utilizing 3D Printing Technology

**Fig. 8:**
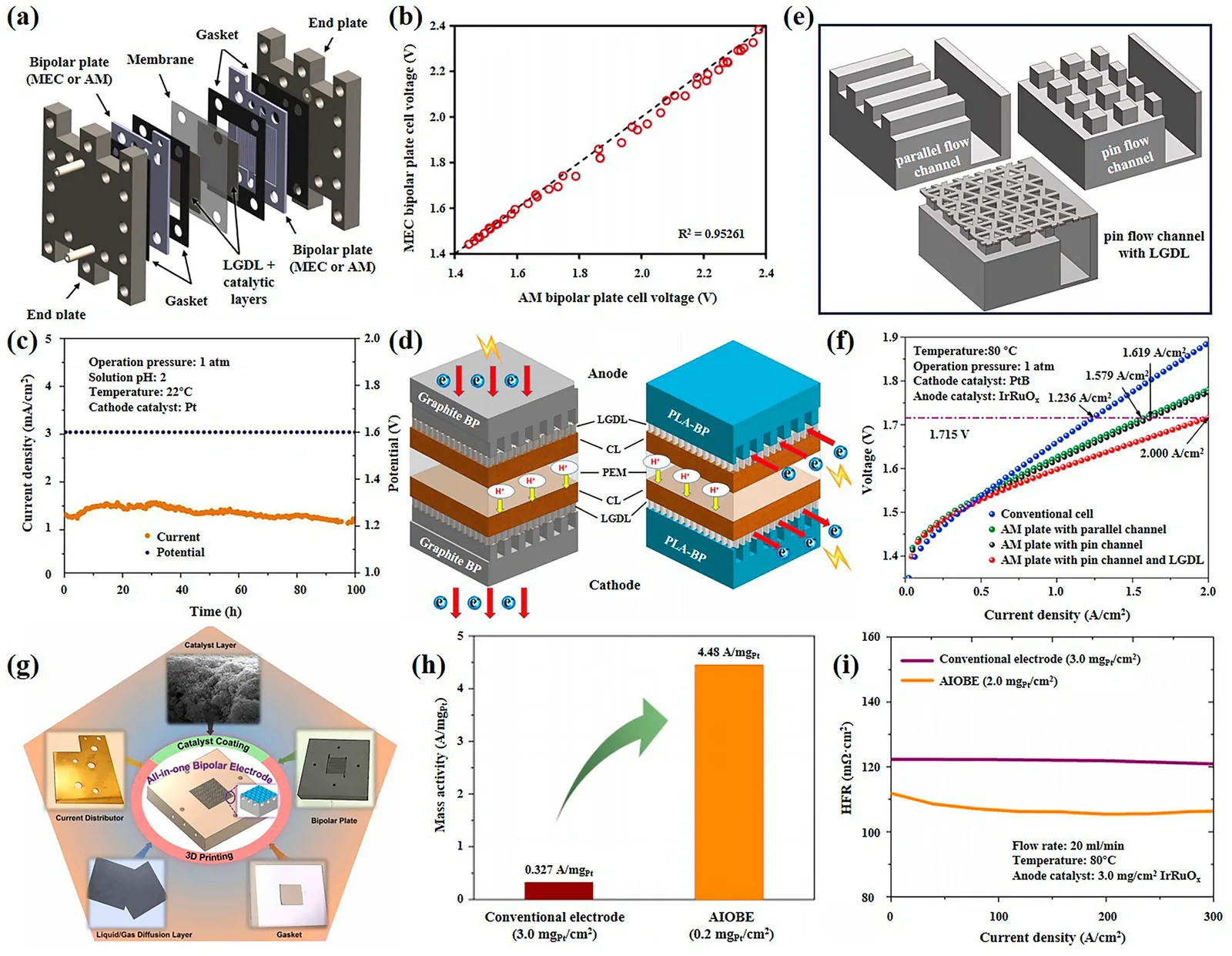
**a** CAD schematic representation of the PEMEC. **b** Parity chart comparing the AM and MEC fabrication methods. Reproduced with permission from Ref. [157]. Copyright 2021, Elsevier. **c** Constant potential polarization curves of the Au-coated AM SS BP at 22 °C over 100 h. Reproduced with permission from Ref. [159]. Copyright 2018, Elsevier. **d** Structural and electronic conduction pathways in traditional PEMEC and novel PLA bipolar plate PEMEC. Reproduced with permission from Ref. [161]. Copyright 2019, Elsevier. **e** Schematics of parallel flow channel, pin flow channel, and pin flow channel with LGDL. **f** Polarization curves of PEMECs with different cathode plates. Reproduced with permission from Ref. [162]. Copyright 2018, Elsevier. **g** Schematic diagram illustrating the 3D-printed flow field and PTL design. **h** Mass activity at 1.6 V for an integrated BP. **i** HFR at 1.6 V for the same integrated BP. Reproduced with permission from Ref. [28]. Copyright 2021, Elsevier

BP serves as a crucial component of the water electrolysis cell, providing structural support as depicted in Fig. 8a. Similar to the PTL, the harsh conditions at the anodic high potentials impose stringent requirements on the material selection for BP, necessitating excellent corrosion resistance and stability. Additionally, to ensure performance, precious metal coatings are often applied, significantly increasing the cost of traditional BP, which can account for over 50% of the total cost of the electrolysis cell. Developing novel, cost-effective fabrication techniques and lightweight materials for BP is a primary method to reduce the costs associated with water electrolyzers. Currently, 3D printing of BP stands out as the most promising technological solution to address this issue [156]. Utilizing 3D printing not only reduces the cost of the plates themselves but also allows for significant improvements in the design of the flow fields on the plates, enhancing the uniform flow of gas and liquid and improving gas evacuation efficiency.

Titanium is commonly used as the metal substrate for manufacturing BP due to its excellent properties, although it is relatively expensive. In an effort to fundamentally reduce costs, Sanchez-Molina et al. [157] utilized laser powder bed fusion technology to fabricate BP from AISI 316L stainless steel (SS), known as additive manufacturing (AM) BP, which were then polished and applied in a PEMEC. Compared to BP manufactured using traditional methods, the AM BP exhibited higher surface flatness, which contributes to reducing interfacial contact resistance. Across all comparison temperatures, the performance of AM BP closely mirrored that of the conventionally manufactured (MEC) BP, showing no significant differences due to the change in material. The correlation between the measured voltages of AM BP and MEC BP reached 0.95261, as illustrated in Fig. 8b, demonstrating the practical application prospects of 3D-printed BP technology. Similarly, using stainless steel as a substrate, Yang et al. [158] produced AM SS BP using SLM technology for application at the PEMEC cathode and conducted the first in situ studies in PEMEC. Notably, at 80 °C, the AM SS BP enabled achieving a current density of 2 A cm^−2^ at a reduced voltage of 1.779 V, outperforming many PEMECs that employ graphite plates on both sides. Yang et al. [159] further investigated the gold plating of AM SS BP. The gold-plated samples were denoted as Au-coated AM SS BP. Under a pressure of 1.45 MPa, the interfacial contact resistance (ICR) of Au-coated AM SS BP was approximately 6.4 mΩ cm^2^, while the ICR of the uncoated AM SS BP was as high as 22.3 mΩ cm^2^. The results indicate that the gold layer can significantly reduce the ICR between AM SS BP and other components, which will help enhance the performance of PEMEC. In addition, to verify the stability of the electrodes prepared by this method, the authors conducted potentiostatic tests on Au-coated AM SS BP in a three-electrode system, as shown in Fig. 8c, achieving excellent durability for over 100 h.

In addition to employing metal materials for the fabrication of BP, the combination of non-conductive materials with localized noble metal coatings has also been explored in BP design and fabrication. Chisolm et al. [160] utilized the FDM technique to print polypropylene BP, which was subsequently enhanced in conductivity by applying two layers of silver paint as a post-treatment. However, due to silver's susceptibility to oxidation, durability testing was conducted to assess the feasibility of this method. Over the 96-h experimental duration, the battery's performance decreased on average by 2.1 mV h^−1^. Specifically, there was a significant drop of 161.2 mV in the first hour of operation. Following this initial degradation, the battery entered a relatively stable phase, with performance declining at a rate of 1.0 mV h^−1^ for approximately 26 h. Thereafter, the battery became more stable, with degradation of only 0.14 mV h^−1^ over the remaining 70 h of the experiment. Although the performance of these BPs still requires improvement to meet the demands of PEMEC, this approach offers novel insights into reducing weight and cutting costs and can potentially be adapted for PEMFC fabrication. In continuous efforts to improve, Husaini et al. [156] developed a polymer BP using DLP, which was further enhanced with nickel and gold coatings through spraying and electrophoretic deposition. Employment of a gold layer increases the conductivity from 300 to 400 S cm^−1^, which enabled the corrosion current density to drop to 0.470 uA cm^−2^. The introduction of a nickel layer not only improved the adhesion of the gold coating but also significantly enhanced the overall conductivity of the BP. However, the actual application and performance of this polymer BP within PEMEC remain unreported, thus necessitating further investigation.

In contrast to traditional flow plates that require the involvement of metallic materials, Yang and colleagues [161] have leveraged 3D printing technology to develop a non-conductive BP, achieving substantial performance breakthroughs. Utilizing the FDM method, they developed a non-conductive polylactic acid (PLA) BP, which eliminates the need for additional post-treatment processes. By employing wet etching to fabricate a novel thin film liquid/gas diffusion layers (TF-LGDL) integrated with the PLA BP, they replaced high-cost technologies. After 100 h of testing, the electrolyzer voltage increased from 1.635 to 1.751 V, with an average decay rate of 1.16 mV h^−1^. However, the decay rate varies over time. Within the first 5 h, the voltage increased from 1.635 to 1.650 V, with an average decay rate of 3.00 mV h^−1^. Over the final 90 h, the electrolyzer reached a stable state, with a decay rate of only 1.03 mV h^−1^. After accounting for PEM degradation, catalyst loss, and oxidation, the negative impact of PLA bipolar plates on PEMEC stability is limited. As illustrated in Fig. 8d, the conventional electron conduction pathway in PEMEC, which typically proceeds through the BP and PTL, is altered in this novel PEMEC design. Electrons are directed laterally from the exterior through the TF-LGDL to the CL, eliminating the need for the PLA BP as a mandatory electron conduction route. This adaptation allows non-conductive PLA to serve as a feasible material for BP, paving a new path for the development of low-cost, high-performance PEMEC and demonstrating the viability of integrating BP with PTL.

Further exploring the capabilities of 3D printing technology, Yang et al. [162] used SLM to integrate four conventional PEMEC components—PTL, BP, gasket, and current distributor—into a single multifunctional AM plate without molds or tools. They experimented with printing different flow structures, including parallel and pin flow fields, successfully printing a pin flow field with PTL, as shown in Fig. 8e. When these AM plates were mounted in the PEMEC for testing, the results were impressive: the pin flow field with PTL demonstrated excellent performance, reaching 2 A cm⁻^2^ at 1.715 V (Fig. 8f). The structural innovations of the multifunctional AM plates provide a fundamental development for the simplification of PEMEC. Shortly afterward, building on the original study, Yang and others [28] proposed the concept of an all-in-one bipolar electrode (AIOBE), which integrates the CL, PTL, BP, current distributor, and gasket into a single component using 3D printing, reducing the number of components in the PEMEC cathode assembly from five to one, as depicted in Fig. 8g. This compact structure significantly enhances the mass activity compared to traditional PEMEC, nearly 14-fold, as shown in Fig. 8h. Even under low loading conditions, the HFR of the AIOBE remains significantly lower than that of traditional PEMEC, as shown in Fig. 8i. This innovative design reduces the number of parts and eliminates ICR, markedly lowering the overall ohmic resistance of the PEMEC, thereby substantially enhancing its performance.

In PEMEC, the design of the flow channels in the BP has garnered significant attention. Currently, due to limitations in manufacturing techniques, the flow channel structures commonly implemented on a large scale include parallel and single serpentine channels. Parallel channels lead to uneven distribution of fluid flow speeds within the channels, adversely affecting the efficiency of electrolysis. On the other hand, single serpentine channels result in longer flow paths and greater flow resistance within the serpentine channels, which can cause uneven flow distribution [163, 164]. Addressing these limitations, the flexibility offered by 3D printing technology enables the customized design of BP. This approach allows for the development of optimized flow channel structures tailored to specific application needs, thereby enhancing electrolysis efficiency. Table 4 summarizes the typical applications of 3D printing in water electrolyzers.

**Table 4:** Typical Applications of 3D Printing in Water Electrolyzers

Specific Applications	Printing Methods	Characteristics of Application	Over-potential	Cell Voltage @1A cm^−2^	Year of Publication	Refs
Electrode	DIW	Ordered periodic controllable 3D electrode	425 mV@1000 mA cm^−2^	/	2020	[145]
	SLS	Ordered coating 3D porous electrode	300 mV@10 mA cm^−2^	/	2022	[151]
	SLM	Microstructured cylindrical electrode	300 mV@10 mA cm^−2^	/	2023	[148]
	DLP	Micro-porous ordered nickel-based electrode	310 mV@500 mA cm^−2^	/	2023	[146]
PTL	EBM	Structurally controllable titanium PTL	/	1.76 V (65 °C)	2016	[154]
	DIW	Graded adjustable ordered nickel PTL	/	1.78 V (80 °C)	2023	[31]
	DIW	Nickel PTL with through-hole 3D periodic structure	/	1.80 V (80 °C)	2023	[155]
BP	FDM	Silver-plated polypropylene BP	/	2.42 V (50 °C)	2014	[160]
	SLM	Gold-Plated SS BP	/	2.32 V (80 °C)	2018	[159]
	FDM	With Titanium Thin film on non-conductive type BP	/	2.21 V (20 °C)	2019	[161]
AIOBE	Metal 3D Printing	Integrated CL/PTL/BP /Current Distributor/Gasket	/	1.62 V (80 °C)	2021	[28]

## Conclusions and Prospects

**Fig. 9:**
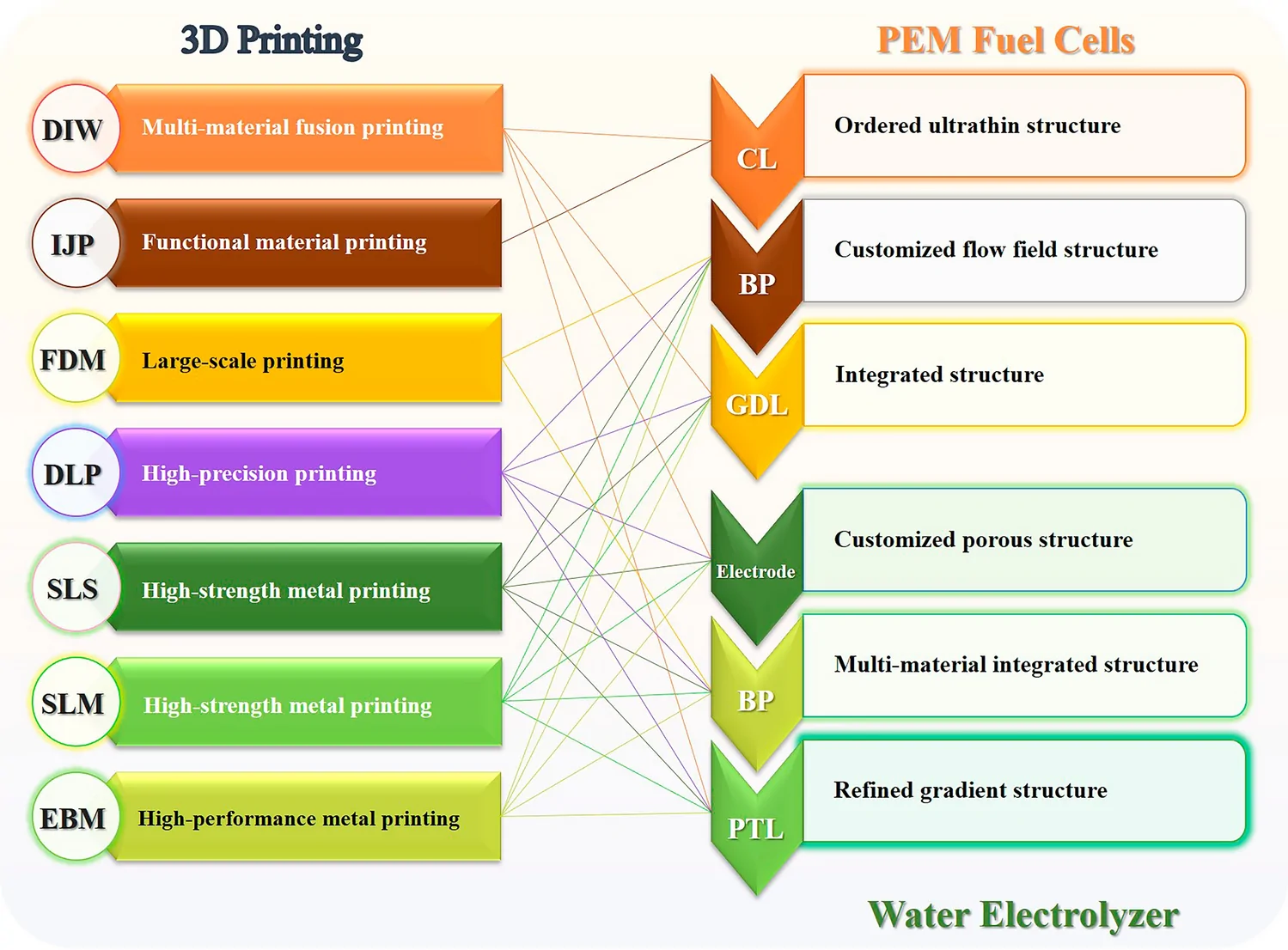
Matching of different 3D printing technologies with PEM fuel cell and water electrolyzer components

3D printing offers notable advantages for hydrogen energy applications, particularly in the fabrication of fuel cells and water electrolyzers. As shown in Fig. 9, the 3D printing technologies that can be matched with PEMFC and water electrolyzer components are summarized. Compared with traditional methods, it provides greater design flexibility, cost control, and production efficiency. Its compatibility with energy device manufacturing makes it a promising tool for structuring key components such as BP, PTL, GDL, and CL. This paper reviews current applications of 3D printing in the hydrogen sector, highlighting its use in designing and fabricating components with improved performance. Metal-based techniques like SLS and SLM are particularly effective, offering organized structures with sufficient mechanical strength, conductivity, and corrosion resistance. However, the limited precision of current 3D printing technologies remains a barrier, especially for the stringent requirements of electrolyzers and fuel cells.

3D printing, as an advanced manufacturing technology that receives significant global attention, is anticipated to overcome its current challenges through dedicated research efforts. The integration of 3D printing with the hydrogen energy sector is viewed as an emerging trend. Based on this perspective, the following projections are made regarding the development of 3D printing in the hydrogen energy domain.

In the development of fuel cells and water electrolyzers, 3D printing technology plays a pivotal role in optimizing key components. In CL, where precision is critical, 3D printing allows for the deposition of ultra-thin, ordered structures, reducing material costs without sacrificing efficiency. One major research focus is the graded and ordered structuring of the GDL and PTL, which significantly enhances material transport—especially under high current densities. Similarly, rational flow field design in BP is crucial for efficient mass transport and stable operation. Simulations combined with 3D printing allow for precise and customizable flow field optimization. For ALK electrodes, 3D printing offers rapid prototyping capabilities, enabling swift adjustments to structure and material configurations with quick experimental feedback. This flexibility shortens the research and development cycle and supports performance improvements. Another emerging trend is the integration of system components to reduce contact resistance, simplify assembly, and lower maintenance and design complexity. 3D printing accelerates the construction and validation of these integrated structures, supporting the development of more compact and efficient devices. Altogether, these advances are driving the evolution of next-generation hydrogen energy systems, opening new pathways for innovation and application in the clean energy sector.

This paper does not cover photolithographic metal manufacturing—a high-precision, material-compatible 3D printing method—despite its growing relevance. This technique supports commonly used substrates in hydrogen technologies, such as titanium and stainless steel, and enables the fabrication of finely structured, corrosion-resistant, and conductive components, all critical to fuel cells and water electrolyzers. As the technology matures, its role in hydrogen energy is expected to grow. Beyond fabrication methods, material development is also key. Corrosion-resistant metal powders are being optimized for better printability and electrochemical durability. Likewise, efforts are underway to design conductive polymers with improved environmental stability, thermal resistance, and mechanical strength—particularly for polymer-based bipolar plates. Additionally, inks with tunable rheology are enabling high-resolution, geometrically complex printing. Together, these material innovations and advanced methods like photolithographic printing are enhancing the performance and reliability of 3D-printed components in hydrogen systems.

Although 3D printing technology is still in the exploratory and developmental stages for applications like fuel cells and water electrolyzers, its potential for advancement in these areas is immense. Continued improvements in 3D printing are expected to drive further innovation in energy device fabrication.

## Abbreviations

3DP: 3D-printed
3DPNi: 3D-printed nickel
ABS: Acrylonitrile butadiene styrene
AEMECs: Anion exchange membrane electrolyzer cells
AFM: Atomic force microscopy
AIOBE: All-in-one bipolar electrode
ALK: Alkaline electrolyzers
AM: Additive manufacturing
BP: Bipolar plates
CCM: Catalyst-coated membranes
CFD: Computational fluid dynamics
CL: Catalyst layers
DIW: Direct ink writing
DLP: Digital light processing
DMD: Digital micromirror device
DMLS: Direct metal laser sintering
EBM: Electron beam melting
ePTFE: Enhanced PTFE
FDM: Fused deposition modeling
GDE: Gas diffusion electrodes
GDL: Gas diffusion layers
HER: Hydrogen evolution reaction
HFR: High-frequency resistance
HOR: Hydrogen oxidation reaction
ICR: Interfacial contact resistance
IJP: Inkjet printing
MEA: Membrane electrode assembly
MPL: Microporous layer
OER: Oxygen evolution reaction
ORR: Oxygen reduction reaction
PEM: Proton exchange membrane
PEMECs: Proton exchange membrane electrolyzer cells
PEMFCs: Proton exchange membrane fuel cells
PLA: Polylactic acid
PTL: Porous transport layers
RDE: Rotating disk electrode
SLA: Stereolithography
SLM: Selective laser melting
SLS: Selective laser sintering
SS: Stainless steel
TF-LGDL: Thin film liquid/gas diffusion layers
TPB: Three-phase boundary
TPMS: Triply periodic minimal surface
UV: Ultraviolet
